# Comparing Algorithms That Reconstruct Cell Lineage Trees Utilizing Information on Microsatellite Mutations

**DOI:** 10.1371/journal.pcbi.1003297

**Published:** 2013-11-14

**Authors:** Noa Chapal-Ilani, Yosef E. Maruvka, Adam Spiro, Yitzhak Reizel, Rivka Adar, Liran I. Shlush, Ehud Shapiro

**Affiliations:** 1 Department of Computer Science and Applied Mathematics, Weizmann Institute of Science, Rehovot, Israel; 2 Department of Biological Regulation, Weizmann Institute of Science, Rehovot, Israel; 3 Department of Biological Chemistry, Weizmann Institute of Science, Rehovot, Israel; 4 Bruce Rappaport Faculty of Medicine and Research Institute, Technion – Israel Institute of Technology, Haifa, Israel; University of British Columbia, Canada

## Abstract

Organism cells proliferate and die to build, maintain, renew and repair it. The cellular history of an organism up to any point in time can be captured by a cell lineage tree in which vertices represent all organism cells, past and present, and directed edges represent progeny relations among them. The root represents the fertilized egg, and the leaves represent extant and dead cells. Somatic mutations accumulated during cell division endow each organism cell with a genomic signature that is unique with a very high probability. Distances between such genomic signatures can be used to reconstruct an organism's cell lineage tree. Cell populations possess unique features that are absent or rare in organism populations (e.g., the presence of stem cells and a small number of generations since the zygote) and do not undergo sexual reproduction, hence the reconstruction of cell lineage trees calls for careful examination and adaptation of the standard tools of population genetics. Our lab developed a method for reconstructing cell lineage trees by examining only mutations in highly variable microsatellite loci (MS, also called short tandem repeats, STR). In this study we use experimental data on somatic mutations in MS of individual cells in human and mice in order to validate and quantify the utility of known lineage tree reconstruction algorithms in this context. We employed extensive measurements of somatic mutations in individual cells which were isolated from healthy and diseased tissues of mice and humans. The validation was done by analyzing the ability to infer known and clear biological scenarios. In general, we found that if the biological scenario is simple, almost all algorithms tested can infer it. Another somewhat surprising conclusion is that the best algorithm among those tested is Neighbor Joining where the distance measure used is normalized absolute distance. We include our full dataset in Tables S1, S2, S3, S4, S5 to enable further analysis of this data by others.

## Author Summary

The history of an organism's cells, from a single cell until any particular moment in time, can be captured by a cell lineage tree. Many fundamental open questions in biology and medicine, such as which cells give rise to metastases, whether oocytes and beta cells renew, and what is the role of stem cells in brain development and maintenance, are in fact questions about the structure and dynamics of that tree. Random mutations that occur during cell division endow each organism cell with an almost unique genomic signature. Distances between signatures capture distances in the cell lineage tree, and can be used to reconstruct that tree. On this basis, our lab developed a method for cell lineage reconstruction utilizing a panel of about 120 microsatellites. In this work, we use a large dataset of microsatellite mutations from many cells that we collected in our lab in the last few years, in order to test the performance of different distance measures and tree reconstruction algorithms. We found that the best method is not the one that gives the most accurate estimates of the mean distance, but rather the one with the lowest variance.

## Introduction

A multi-cellular organism develops from a single cell – the zygote, through cell division and cell death, and displays an astonishing complexity of trillions of cells of different types, residing in different tissues and expressing different genes. The development of an organism from a single cell until any moment in time can be captured by a mathematical entity called a cell lineage tree [1]–[4].

Uncovering the human or even the mouse cell lineage tree may help to resolve many open fundamental questions in biology and medicine, as illustrated by our earlier work [5]–[9].

In the past few years, our lab developed a method for reconstructing the lineage relations among cells of multi-cellular organisms 1,10 and applied it to various questions of biological and medical importance [5]–[9]. The method is based on the fact that cells accumulate mutations during mitosis in a way that, with a high probability, endow each cell with a unique genomic signature, and distances between genomic signatures of different cells can be used, in principle, to reconstruct the organism's cell lineage tree [1]. Instead of examining the whole genome of all cells of an organism, which is currently not feasible, our method uses Microsatellite (MS) loci which are repeated DNA sequences of 1–6 base pairs. Slippage mutations, in which repeated units are inserted or deleted, occur at relatively high rates (10^−5^ per locus per cell division in both wild type mice and humans [1], [11]), and thus provide high variation. These mutations are phenotypically neutral [11]–[13] and they are highly abundant in the genome (composing 3% of the genome). Importantly, Mismatch-Repair (MMR) deficient mice display an even higher mutation rate (10^−2^ per locus per cell division [14]) in MS and are available for experimentation and analysis [5]–[8], [10], [15], [16]. By comparison, SNPs have a mutation rate of the order 10^−8^ per site per generation [17], and thus about 10^−10^ per site per cell division.

Besides the use of MS to reconstruct cell lineage trees which was proposed also by others [3], [4], [18]–[20], several other retrospective methods to trace cell lineages in mammalians have been proposed. These methods include the genomic profiling of single nucleotide polymorphism (SNP) [21]–[23], copy number variations (CNV) [24] and DNA methylation [25]–[27]. A common feature of all these methods is the use of a genomic property that accumulates mutations during cell divisions, thus making it suitable to be used as a genomic signature.

While phylogenetic lineage tree reconstruction of cells is similar to that of organisms and species, it also has unique characteristics such as the existence of stem cells (that influence the shape of the tree), a sometimes very shallow tree (in the order of dozens of generations), a dramatic variation in the number of divisions the cells have undergone since the zygote (which is much larger than what exists in species with different evolutionary paces), and the fact that the cells have undergone binary cell divisions. Besides the last feature, which has been widely investigated in population genetics [28], these unique characteristics as well as the uncertainties about the exact nature of the mutational process in somatic cells require an assessment of the accuracy of known lineage reconstruction algorithms for this application. Another meaningful difference is that MS are usually used to define relationships between groups (species or populations) [29], and not between individuals. Thus the mathematical measures defining the distance are different.

The goal of this work is to test the existing algorithms on experimental cell data and to validate their use. In addition we want to test which of these methods (even though not developed for the purpose of cell lineage reconstruction) performs best.

In order to accomplish this goal, we took experimental data from clearly known biological expectations and examined whether the reconstructed trees present this knowledge. The data was obtained by isolating cells from different mice and humans, and extracting their genomic signatures (see Materials and Methods). Due to the fact that the real cell lineage tree is not known, we examined two aspects of the estimated tree. One aspect is the clustering of biologically distinct cell groups on the tree, and the second is the ability to distinguish between two groups of cells that are known to have different *depths* (number of divisions the cell has undergone since the zygote).

## Results

### Validating and quantifying the ability to reconstruct a cell lineage tree

As mentioned earlier the goal of this work is to validate and quantify the ability to reconstruct a cell lineage tree utilizing genomic signatures of individual cells that record mutations in microsatellites. Since precise inference of tree topology cannot be accomplished using our current limited number of loci (See Table S6), we examined in this work whether certain aspects of the inferred tree reflect known biological scenarios. The first is the clustering of different cell groups. The basic assumption of this test is that if a statistically significant clustering on the cell lineage tree is consistent with a biological characteristic, then such clustering is very likely to reflect a real biological phenomenon, and therefore the more significant the clustering found by an algorithm, the better the algorithm. The simplest possible grouping of cells can be according to which individual they belong. Investigating the lineage relations among cells of different individuals is normally not done, however it is useful as a benchmark to test the validity of cell lineage reconstruction algorithms, as cells from different individuals clearly should be clustered separately. The second aspect we examined is the depth separation between different types of cells that are known to have different depths.

The tree reconstruction algorithms that we used are Neighbor Joining (NJ) [31], UPGMA [32], and a quartet-based method as implemented in the QMC tool [33]. The distance measures that we used are two versions of the Absolute genetic distance (regular and normalized), Euclidian distance, Equal or Not distance, and six versions of likelihood distances – assuming equal mutation rates for all loci, assuming two different mutation rates for mono-nucleotide and di-nucleotide repeats, and assuming length dependent mutation rates. These three mutation models were tested on both the Stepwise Mutation Model – SMM, and the Multistep Mutation Model – MMM (for more details regarding the reconstructing methods see Materials and Methods). In addition to distance-based algorithms, Bayesian methods can also be used to infer the cell lineage tree. Even though these Bayesian methods hold great promise, there are currently only a very limited amount of existing tools that can be used to analyze MS. In addition most of the existing tools (such as MrBayes [34], [35], Migrate [36] and Beast [37]) assume no linkage between the different loci. However, in the cell-lineage tree, the range of the linkage disequilibrium is infinite (i.e. the whole chromosome is fully linked, as the mitotic recombination rate is very small compared to the MS mutation rate multiplied by the chromosome length and the depth of the trees). Moreover, in the special case of cells inside multicellular organisms, since each cell has only one single parent cell from which all its chromosomes derive, all the MS loci share the same history, and hence all the loci are fully linked (including loci that are on different chromosomes).The only ML tool that we found to be applicable to our case is BATWING [38] which reads in multi-locus haplotype data, a model and prior distribution specifications. It may be worthwhile to test the performance of the other Likelihood\Bayesian algorithms, even though they assume that loci are not fully linked; however, these algorithms are highly computationally intensive, and therefore we could not test these.

### Clustering

**Figure 1:**
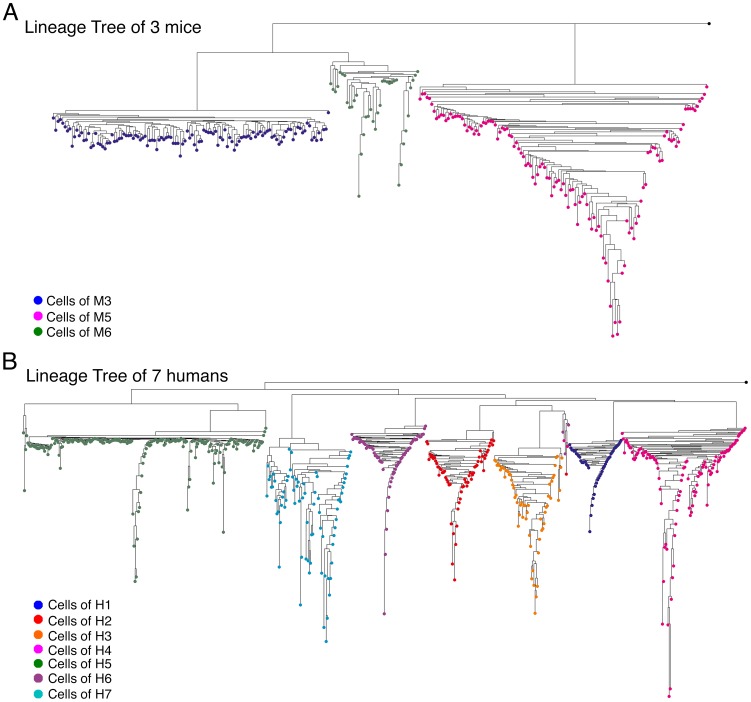
Cells from different organisms are clustered separately in the lineage tree. Two reconstructed lineage trees are shown: (A) A lineage tree containing cells from three mice, M3 (blue), M5 (pink) and M6 (green). (B) A lineage tree containing cells from seven humans, H1 (blue), H2 (red), H3 (orange), H4 (pink), H5 (green), H6 (purple) and H7 (turquoise). The root of the trees (colored in black) is the weighted mean of the organisms' putative zygotes. The trees were reconstructed using the NJ algorithm along with the Normalized-Absolute distance measure.

In this section we checked the clustering quality of the tree reconstruction methods. An example for a case where cells from different individuals are clustered distinctively on the tree can be seen in Figure 1. We used all cell types (see Table S1 for the list of cell types of each individual) from three mice (Figure 1A) and from seven humans (Figure 1B), with the Normalized Absolute genetic distance and the Neighbor Joining tree reconstruction algorithm. It can be seen that the cells of each individual are clustered separately on the tree.

However, due to the many types of noise existing in the system, such a distinct clustering is not likely to happen in all cases, especially if the individuals are related to each other (as in the case of some of the experimental mice), and their zygotes are genetically close. In such cases, due to the small panel of MS used, cells from different individuals can randomly accumulate mutations that reduce the genetic distance between them, and may become closer to each other than to other cells from the same individual. This effect depends on the ratio between the genetic distance between the zygotes of the mice and the number of divisions the cells in each mouse underwent. We showed via computer simulation (Text S1 and Figures S1, S2, S3) that when this ratio is small it is very hard to distinguish which cells belong to which mouse. In addition, we showed that as the number of loci grows, the separation between the mice improves.

**Figure 2:**
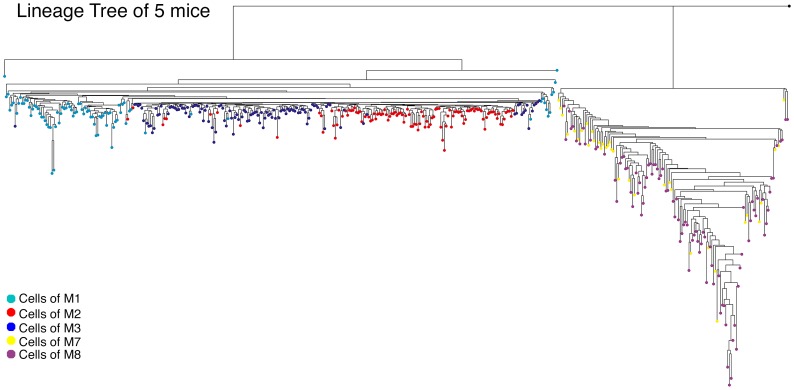
Cells from different organisms are mixed in the lineage tree. A reconstructed lineage tree containing cells from six mice, M1 (turquoise), M2 (red), M3 (blue), M7 (yellow), and M8 (purple). The root of the trees (colored in black) is the weighted mean of the organisms' putative zygotes. The trees are reconstructed using the NJ algorithm along with the Normalized-Absolute distance measure.

Our panel contains ∼120 loci but we prefer to ignore loci where there are allelic dropouts- i.e. where there is amplification failure of one of the two heterozygous alleles while the other allele successfully amplifies, which may often be misinterpreted and lead to errors in allele size determination. We thus used an average of about 80 loci per cell. In addition, those 80 loci can be different between distinct cells, so the actual number of loci used for the distance calculation can be even smaller (with a minimum limit of 25). We showed that when the ratio is 0.2 and the mutation rate is 1/100, a separation of 90% can be achieved using at least 200 loci. An example of such a case is shown in Figure 2 where we present the tree of five mice with all their cells. It can be seen that three of these mice (M1, M2 and M3) are separated quite well in the lineage tree, compared to the cells of the other two mice (M7 and M8) which are strongly mixed. It may be due to their possible family relations; however since we do not know the real relations but rather estimation, other types of noise can mix the cells, such as errors in the amplification of the genetic sequence or in the PCR reaction.

**Figure 3:**
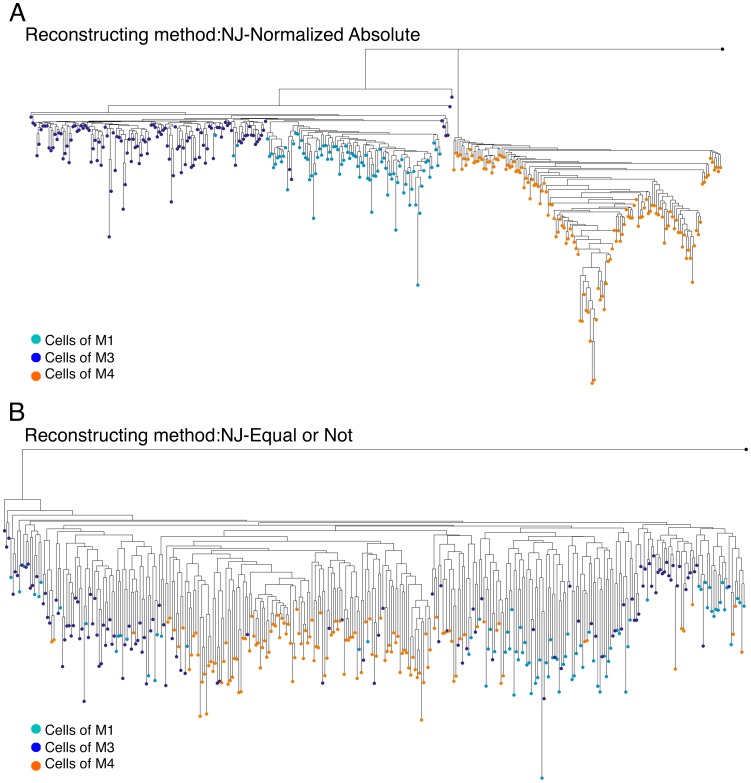
Performance of different methods on the same dataset. Two reconstructed lineage trees containing the same cells from three different mice are shown: M1 (turquoise), M3 (blue), and M4 (orange). (A) A tree using the NJ algorithm along with the Normalized-Absolute distance measure. (B) A tree using the NJ algorithm along with the Equal or Not distance measure. The root of the trees (colored in black) is the weighted mean of the organisms' putative zygotes. It can be seen that the performance of the Normalized-Absolute distance measure is clearly better.

Nevertheless, not all the algorithms and distance measures suffer from this problem to the same degree. This may be due to the fact that some measures describe the mutational process more accurately, or due to some other robustness feature of the algorithm. An example for a different performance between different metrics is shown in Figure 3 where we applied two methods to the same dataset. It can be seen that the NJ- Normalized Absolute (Figure 3A) produced better cluster separation than the NJ- Equal or Not method (Figure 3B).

The difference between methods in the clustering separation of cell groups necessitates the quantification of their performance, in order to determine which method is the best (if any). In order to do so, we used three measures to quantify the clustering quality of distinct groups: the Quality of the Largest Cluster (QLC) from each group, the Tree Entropy (TE), and the probability of getting such a cluster under the assumption of hyper geometric sampling (HS). (A detailed explanation of the measures is given in the Materials and Methods).

We analyzed the performance of the methods on all the information that we have available: cells from nine mice and seven humans, each containing a few types of cells (Table S1). We combined two or more individuals into one larger dataset, using all their cell types or a single type. For each dataset, we reconstructed the cell lineage tree using all the methods we have. Then we quantified the separation performance of each method with the measures listed above, and determined the method with the best performance. In the following section, we present the results of this test and its variants.

#### All cells together

We started with rough wide-scale tests, reconstructing all the optional combinations of the datasets, regardless of whether different individuals share the same types of cells. We then continued with more refined tests, which pay attention to the specific types of cells, and used datasets containing only one type of cell. From the nine mice that we have, we could produce 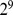 datasets but since they contain sets of zero or one individual and these are not relevant to our analysis, the number of datasets is 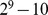 . We reconstructed the cell lineage tree of each dataset using NJ and QMC algorithms, and quantified the clustering separation between the different mice using the three measures. UPGMA and BATWING assume that all the cells have the same depth, and although we test these methods for all cases, only those cases where the similar depth assumption is reasonable, are interesting.

Examples of the results on a few specific datasets are presented in Table 1. These datasets present cases where all (or almost all) the methods successfully separate the cell groups; cases in which all the methods do not perform so well, and cases where a few methods perform well while others fail. (Examples of all the reconstructed trees of a few specific datasets are presented in Figures S4, S5, S6).

**Table 1:** Example of results on a few datasets.

#	Name	Score	NJ-	NJ-	NJ-	NJ-	NJ-SMM	NJ-SMM	NJ-SMM	NJ-MMM	NJ-MMM	NJ-MMM
			ABS	NormABS	EqualOrNot	EUC	equal rates	monoDi rates	lenDep rates	equal rates	monoDi rates	lenDep rates
**1**	M1M2M3M4M5M6M7M8M9	QLC	0.347823	0.275611	0.37813	0.301447	0.371728	0.350756	0.368669	0.365139	0.30166	0.287746
	All cell types	TE	84.40917	60.78266	101.2351	97.113	92.00133	92.45571	122.3109	102.0853	93.23476	112.3511
		HS	0	0	0	0	0	0	0	0	0	0
**2**	M1M3M4M5M6	QLC	0.682521	0.705587	0.496068	0.487241	0.703336	0.610997	0.633189	0.723407	0.731626	0.674685
	All cell types	TE	70.22079	67.6069	138.9407	83.75034	94.94297	108.827	115.5496	98.87133	95.21636	95.26127
		HS	0	0	0	0	0	0	0	0	0	0
**3**	M1M2M6	QLC	0.753549	0.945355	0.553541	0.633164	0.78515	0.74055	0.674994	0.638117	0.642526	0.802127
	All cell types	TE	105.9908	14.81306	201.029	250.6545	147.167	103.9354	116.4198	170.2311	163.8052	96.83945
		HS	0	0	0	0	0	0	0	0	0	0
**4**	M1M2M3M9	QLC	0.522825	0.761499	0.353106	0.364581	0.568366	0.478402	0.520778	0.497532	0.498754	0.522246
	All cell types	TE	234.5802	98.08597	296.6947	333.6574	216.7348	254.1092	202.1498	237.8789	218.5638	205.0351
		HS	0	0	0	0.000288	0	0	0	0	0	0
**5**	M1M4	QLC	0.805541	0.99087	0.676715	0.738966	0.811159	0.811159	0.996552	0.996552	0.811159	0.996552
	All cell types	TE	98.5246	10.80349	366.0483	253.7946	128.6547	97.8306	5.655992	5.655992	102.336	5.655992
		HS	0	0	0.000127	0	0	0	0	0	0	0
**6**	M1M9	QLC	1	1	0.747105	0.841311	0.988636	0.977273	1	1	1	1
	All cell types	TE	0	0	125.1425	157.2069	4.430817	8.501064	0	0	0	0
		HS	0	0	0	0	0	0	0	0	0	0
**7**	M5M9	QLC	1	1	1	0.899543	1	1	1	1	1	1
	All cell types	TE	0	0	0	87.94826	0	0	0	0	0	0
		HS	0	0	0	0	0	0	0	0	0	0

The table represents the results of the three clustering measures (QLC, TE and HS) over 10 NJ reconstructed methods. For the measure QLC higher scores mean better performance, whereas for the measures TE and HS lower values mean better performance. It can be seen that in some cases, for example dataset #7, all the distance measures (except for Euclidian) give the same best score (1 for QLC and 0 for TE and HS). For dataset #1, on the other hand, all the distance measures give a rather similar bad score. It is not surprising that the scores of a dataset which contains 9 individuals (#1) will be lower than the scores of a dataset which contains only 2 individuals (#7). There are cases, like #3 and #4, in which the best performance is achieved by one method (NJ-Normalized Absolute) and other cases, like #5, where a few methods receive a very high score (NJ-Normalized Absolute, NJ-MMM length dependent rates, and more).

**Figure 4:**
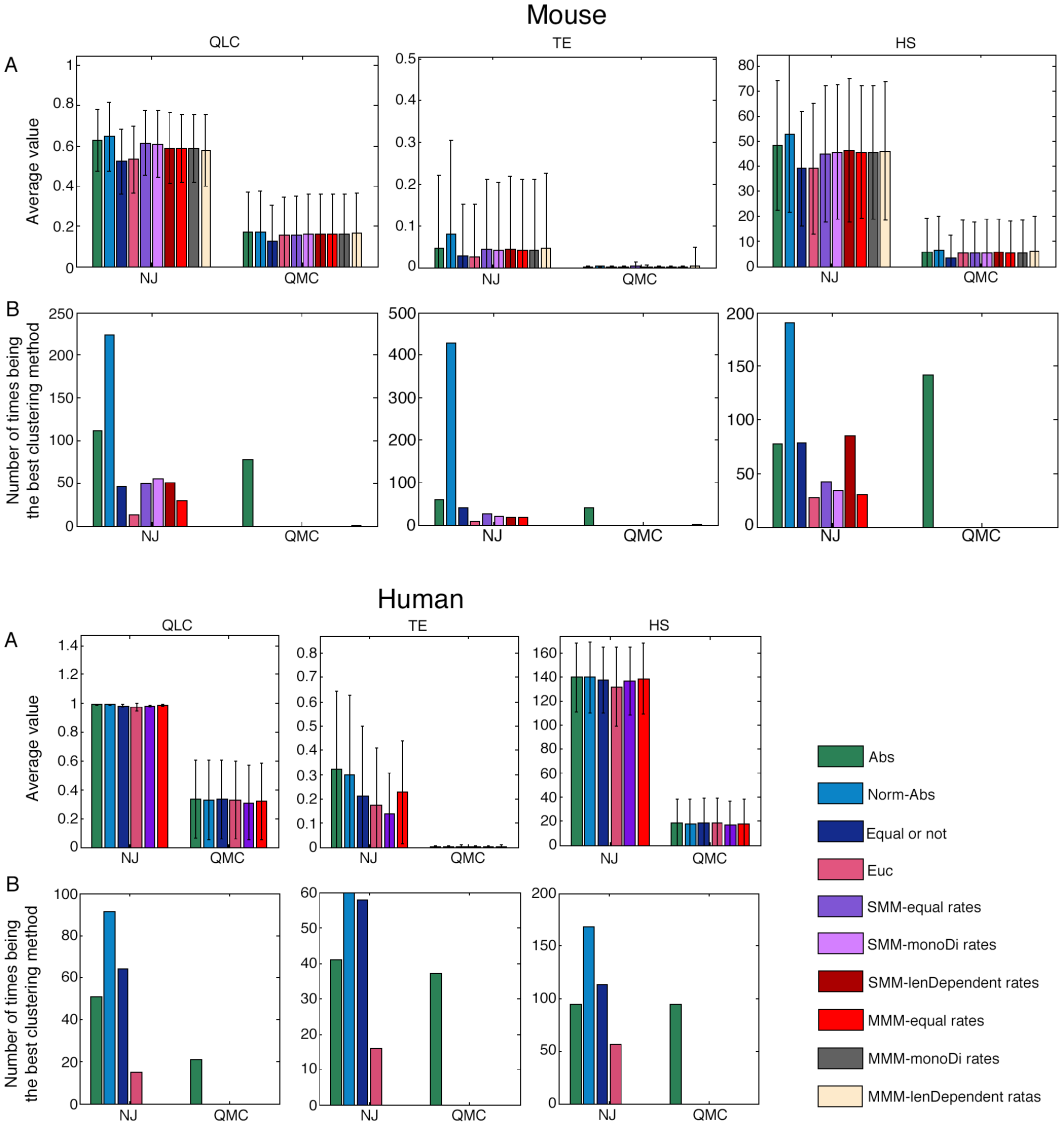
Performance summary of all the methods on all the datasets of mice and humans. Upper panels – Mouse, Lower panels- Human. Each column presents a different clustering measure (see Materials and Methods for details), and each bar represents a different distance measure, where the colors specify the distance measures as noted in the legend. The first and the second group of bars (from left to right) present the results using the NJ algorithm and the QMC algorithm respectively. Rows description: (A) The average score of all the methods, where higher values (that are transformations of the real scores) indicate better performance. (B) The number of times every method received the highest rank (for the mouse panel the highest rank is 20 since we compared 20 methods, and for the human panel the highest rank is 12).

The results of the 502 datasets are given in Table S5.1. A summary of the results is given in Figure 4 (upper part). The upper panels of the figure (Figure 4A) present the average score of all the methods. The values are transformations of the real ones such that higher values mean better performance. (For detailed explanations of the measures and the transformations see Materials and Methods). The second panels (Figure 4B) show the number of times every method got the highest rank (in this case it is 20 since we compared 20 methods: 10 NJ and 10 QMC methods). It is important to emphasize that more than one method can be ranked a specific rank; this is when two or more methods get the same score. The results of the two other algorithms (UPGMA and BATWING) as well as other representations of the results are shown in Figure S7, where we show the normalized average scores in which the higher the values the higher the performance. This representation is needed in order to enable a comparison of the results of ‘simple’ and ‘complicated’ scenarios simultaneously. In simple scenarios, each group of cells is strongly separated so all the methods receive a good score, while in complicated scenarios the cells are mixed and none of the methods' performance is good. Another representation is the average rank of each method, where a method receives the highest rank when its score is best.

As seen, the NJ- Normalized Absolute method is ranked the best more frequently than the other methods in all the three different measures, and the next best performance is associated with the NJ- Absolute method. The absolute distance measure captures the underlying mutational process precisely only if there are no back mutations, namely the addition of a repeat followed by its deletion, or vice versa. However, this is not likely to be the case in general as empirical data shows that MSs accumulate many mutations and yet have small variability over time, hence back mutations must be present, most likely in accordance with a random walk model [39]. We believe that normalizing the mutations (as in the Normalized Absolute measure) eliminates the depth of the cells and therefore the weight of the loci with fewer mutations is larger, and neglecting the backward mutations has less effect.

We conducted a similar rough test (i.e. including different types of cells) using the human data which contains seven individuals, thus producing 120 datasets. A summary of the results is given in Figure 4 (bottom part) and Figure S7 (full results are given in Table S5.2). It can be seen that all the distance measures along with the NJ algorithm give an almost perfect separation, however the NJ-Normalized Absolute still performs best, just as with the mice datasets. Note that for the human datasets we compared only two ML methods (and not six), as we do not have an estimation of different mutation rates for mono-nucleotide and di-nucleotide repeats.

In order to validate these results we performed lineage tree reconstruction of simulated trees similar to the ones reconstructed using the real data (as described in Materials and Methods). The simulated trees contained cells separated according to their types and the individual to which they belong. We simulated several topologies in which three depth parameters were varied: 1. the distance between the root of the tree and the individuals' zygotes, 2. the distance between the individual's zygote and the MRCA of each cell type group, and 3. the depth of each cell (examples are shown in Figure S8). The simulations show that when the distance between the leaves and their MRCA (most recent common ancestor) is high, compared to the distance between the root of the simulated tree and these MRCA (topology A in Figure S8), using ∼100 loci (the amount used in the experiments analyzed in this paper), the Normalized-absolute distance performs best in separating the different cell types. This result agrees with our analysis of the real data (results are shown in Table S7.1 and in Figure S9A). Using more than 100 loci or less, similar results are obtained, whereas when the amount of loci increases, the performance increases as well. However, when the distance between the leaves and their MRCA is small, compared to the distance between the root and these MRCA (topology B in Figure S8), only for small mutation rates (1/10000) does the Normalized-absolute distance perform best. For the other cases, it is difficult to conclude which method is best.

#### Cells with similar depth

In general one cannot assume that the depth is the same for different cell types, and therefore tree reconstruction algorithms that produce ultrametric trees are not applicable. Even though, by using the same type of cells from organisms with the same age, the same depth assumption is reasonable, still, there might be a problem that the zygotes of the different organisms do not have the same depth. However due to the fact that our mice are MMR deficient, their mutation rates after the zygote are a few orders of magnitude higher than before the zygote, and all of them are genetically related, it is reasonable to assume that they have the same depth from the zygote.

**Figure 5:**
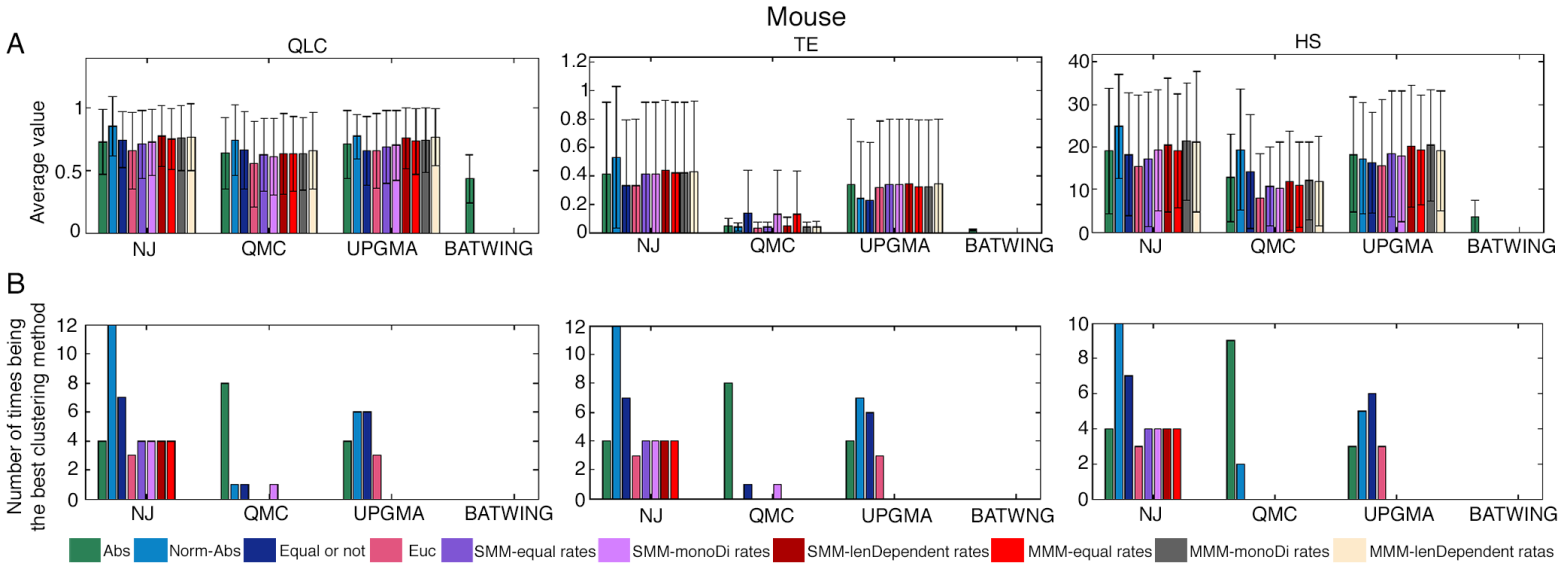
Performance summary of all the methods for datasets composed of cells of same depth only. Each column presents a different clustering measure (see Materials and Methods for details), and each bar represents a different distance measure, where the colors specify the distance measures as noted in the legend. The first group of bars (from left to right) presents the results using the NJ algorithm, the second group of bars presents the results using the QMC algorithm, the third presents the results using the UPGMA algorithm, and the last one presents the results using the BATWING tool. Rows description: (A) The average score of all the methods, where higher values (that are transformations of the real scores) indicate better performance. (B) The number of times every method received the highest rank (in this case it is 31 since we compared 31 methods).

By using the same cells from different mice with the same age, we also tested the UPGMA algorithm with all the distance measures that we have, as well as BATWING. In general the NJ and the UPGMA algorithms perform better than the QMC and the BATWING. The NJ has a small advantage over the UPGMA, but since the dataset size is relatively small we cannot provide a convincing conclusion. Among all the measures, using NJ- Normalized Absolute performs the best as shown in Figure 5 and Figure S10 (full results are given in Table S5.3.). In addition, both UPGMA and QMC (in some of the clustering measures) perform similarly to NJ, however using these algorithms the Normalized Absolute distance is not always the best but also some of the ML distances.

The Bayesian method did not perform well, compared to the other methods. This is not surprising taking into account the fact that its assumptions do not describe well the cell population, both in the reproduction process, and the demographic model. BATWING assumes an exponential growth, while the true demography is much more complicated and includes for instance bottlenecks for the different types of cells [6]. This indicates that in order to apply Bayesian and Likelihood methods to cell populations, there is a need to develop specific tools that will fit cell populations.

#### Many cell types in one individual

The comprehensive test presented above is not identical to the common cases in cell lineage research. In the above test, we used cells from different organisms, while in the common research questions of cell lineage, cells from the same organism are used. A priori there is no reason to believe that the evolvement of the mutational process inside the individual is different than the one between different individuals. This is because many general features of the MS mutational process (such as the dependency on the unit size, the length of the MS, and the specific letter) show a similar behavior (Chapal-Illani et al, in preparation), and because the MMR-deficient mice are relatives, and thus the genetic distance between them is relatively small and the vast majority of their mutations are somatic. As a result we assume that there is no difference in the performance of the reconstructing algorithms, and since the NJ-Normalized Absolute algorithm performs best between individuals, we can assume it has the best performance inside a single individual. To validate this assumption we reconstructed lineage trees of single cells from the same individual, using all types of methods mentioned above, without knowing what the true separation of the different cell types is. In Figure S11 we present the performance of the methods on a relatively small sample: eight mice and seven humans that have more than one type of cells. For each mouse and human we used all the cell types that we have (see Table S1 for the list of cell types of each individual). Even though the real scenario is not clear, we found that there is a separation between the cell types, and the method with the best performance (but not substantially better) was the NJ-Normalized Absolute, similar to the results obtained for different individuals. We performed a permutation test which involves the shuffling of the different cells in the reconstructed tree, and we found that the separation between the cell types is statistically significant (P<10^−3^). That indicates that the separation we observed is not random and we can assume that the method for separating between individuals works just as well for separating within an individual.

We also performed lineage tree reconstruction of simulated trees of single cells of different types from one individual (described in Materials and Methods). We simulated several topologies, similar to the ones we performed for the simulated trees, containing several individuals (examples are shown in Figure S8). The simulations showed that the NJ-Normalized Absolute has an advantage over the other method. However, this holds when the distance between the leaves and their MRCA is high, compared to the distance between the root of the simulated tree and these MRCA (topology A in Figure S8). When the distance between the leaves and their MRCA is small (topology B in Figure S8), the performance of NJ-Normalized Absolute is not always the best. These results (Table S7.2 and in Figure S9B) also agree with the simulations of the trees containing multiple individuals that we performed.

#### Same type of cells

In order to examine the clustering abilities in a refined way, we produced datasets using only one type of cells from different individuals, and tested the performance of the different methods. This step is important for two reasons: a. The use of different cell types for different mice may lead to an artificial separation between the mice, simply because of the diverse features of different cells (such as division rate, etc.). The use of the same type of cells assures that the methods indeed distinguish between the different organisms. b. We want to check the presence of a correlation between the clustering qualities of the different methods with specific types of cells, i.e. whether there are some methods that succeed in separating some types of cells, while others succeed in separating other types. Figure S12 shows the results of the methods performed on 57 mouse datasets and 146 human datasets. Overall, the results are consistent with the results obtained by using various cell types together indicating that the NJ-Normalized Absolute is the best method.

### Depth separation

As mentioned before, clustering is just one example of a feature of the tree in which we are interested. Another feature is the depth of specific types of cells. Different depths can indicate different biological scenarios, for example whether some types of cells divide only during the embryonic stage or also in the adult stage. In this section our goal is to quantify the performance of the different methods in identifying depth differences between groups of cells.

**Figure 6:**
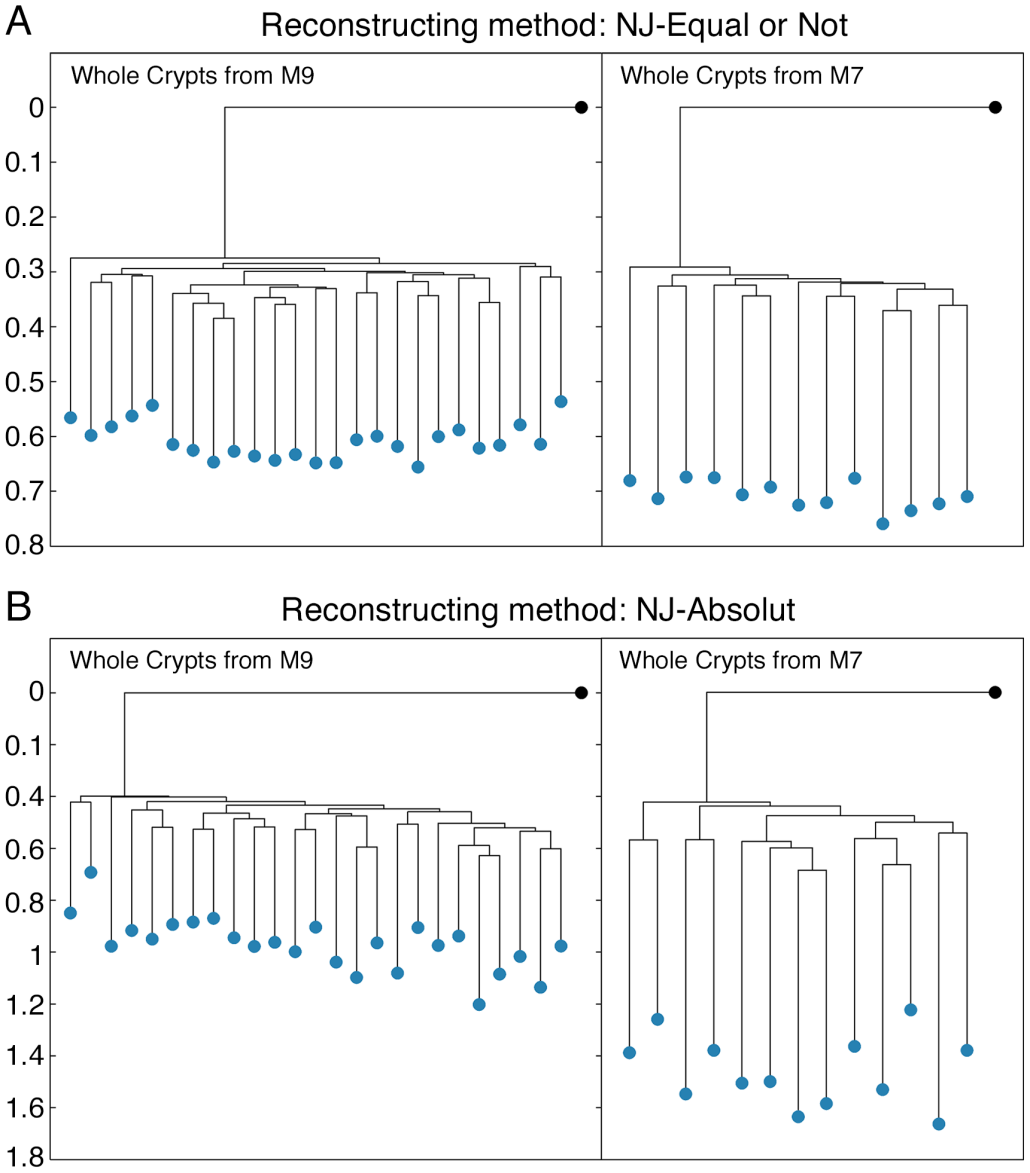
Depth separation comparison of two distance measures on the same dataset. Two reconstructed lineage trees containing whole crypts from M9 (52 days) and two reconstructed lineage trees containing whole crypts from M7 (199 days) are shown. The root of the trees (colored in black) is the signature of the tail extracted from each mouse. (A) Two reconstructed trees using the NJ algorithm along with Equal or Not distance measure. (B) Two reconstructed trees using the NJ algorithm along with the Absolute distance measure.

In order to obtain cases where the depth separation between the cell groups is known, we used the same type of cells from individuals with a substantial gap between their ages. The list of cell types of each individual is given in Table S1. The tree-reconstruction algorithm that was applied in this case is the NJ algorithm, since it is the only algorithm that allows different depths for different cells inside the same individual. Two examples of trees with depth difference are presented in Figure 6, one with a good depth separation (Figure 6B) and one with a poor separation (Figure 6A).

The depth of each group of cells as reconstructed by the NJ varies even if all the cells of this group actually have exactly the same number of divisions. Therefore for each group of cells, the depth is described by a distribution rather than by a single number. In order to quantify the performance of a method in separating between the two groups, quantities that differentiate between distributions are needed.

The most natural choice is the Kolmogorov-Smirnov (KS) test, which measures the similarities between two datasets. However, this test has some disadvantages for our purposes; the most significant one is the ability to determine that two datasets are different even if they have exactly the same average depth, in cases where their standard deviations are substantially different. Therefore in addition to the KS we added two other measures that focus on the separation between the two distributions. The first is the normalized distance between the mean of the two groups, and the second is the overlap percentage between the two distributions (see Materials and Methods for more details). The difference between them is that the overlap percentage is affected by the behavior of the extreme cells, while the normalized average distance captures the behavior of the bulk. Another minor difference is that the average normalized distance can distinguish between the separation qualities of methods even in the case of fully separated groups.

**Figure 7:**
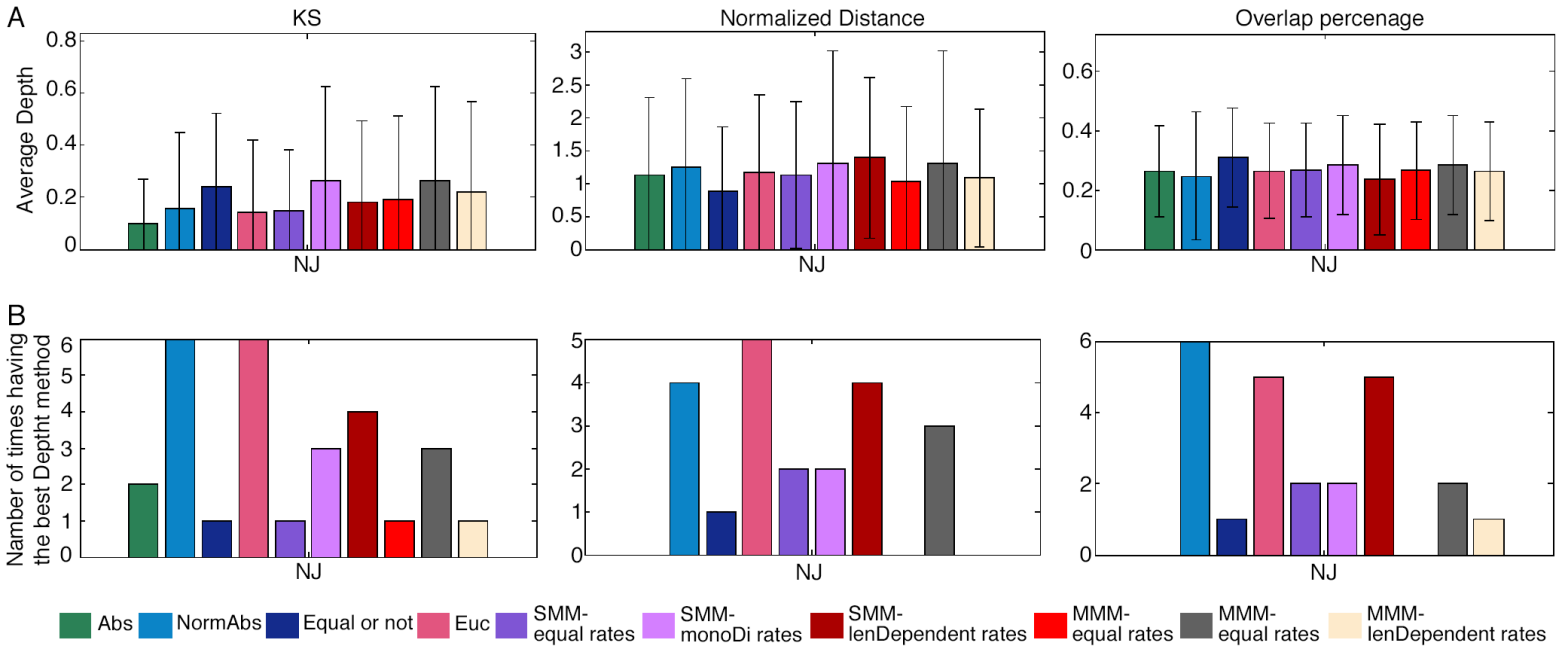
Depth quality of the different methods. Each column presents a different depth quality measure (see Materials and Methods for details), and each bar represents a different distance measure, where the colors specify the distance measures as noted in the legend. Rows description: (A) The average depth score of all the methods, where lower values for the KS test, lower values for the overlap percentage and higher values for the normalized distance test mean better performance. (B)The number of times every method received the highest rank (in this case it is 10 since we compared 10 methods).

A summary of the depth separation tests' results is presented in Figure 7 and Figure S13 (full results are given in Table S5.4). In this case there is no one method which is superior over the others, but a few which are rather equally good: Normalized Absolute, Euclidean and SMM with length dependent mutational rates. This implies that for the depth separation there is no one tree which can be considered the correct one. The various inferred trees should be seen as approximate projections of the real tree, which cannot be inferred precisely as of yet, since the genetic identifier is not sufficiently informative. These results were validated by simulations in which lineage trees with different cell depths were reconstructed and the differences were evaluated using the depth measures described in Materials and Methods. In each iteration, two lineage trees were simulated and the difference between the depths was calculated, where we distinguished between cases in which the trees were relatively shallow, and cases in which the trees were relatively deep. The simulations show that when the trees are shallow, when using 100 loci, there is no one method which is uniformly better than the others, in accordance with our result obtained with real data. When using 50 loci, the Normalized-absolute has the worst performance, while with 500 loci; the Normalized-absolute performs best. When the trees are deep, with 500 loci there is no one method that is better than the others, whereas when using fewer loci, the Normalized-absolute has the worst performance (results are shown in Table S8 and in Figure S14).

### Reliability of reconstructed lineage trees

Bootstrap analysis was used in order to evaluate the robustness and reproducibility of the estimated trees, the clustering of the tree and the depth separation according to cell type. We performed this analysis on several mice and human datasets which showed a good clustering or depth separation using the NJ-Normalized Absolute method.

The bootstrap values were obtained by generating 100 trees using MS values extracted from sampling with replacement of the loci from each dataset. The bootstrap showed that the robustness of any particular branch in the tree is low, but the robustness of the clustering results and depth separation according to cell type is high (see Table S9 for all the results).

## Discussion

In the preliminary stages of the cell lineage research conducted by our lab, small-scale investigations of the ability to reconstruct cell lineage trees were done. The large amount of information that was gathered during the last few years enabled us to conduct this investigation in a much more comprehensive way. The main outcome of this research is that even though currently only a limited amount of microsatellite loci are available, preventing the reconstruction of the accurate cell-lineage tree, many biological conclusions can still be confidentially drawn. By this we mean that apart from specific noisy cases, almost always we are able to identify the correct biological scenario from the reconstructed cell lineage if the proper tree reconstruction algorithm and distance measure are used.

Among the NJ methods, we have found that the NJ- Normalized Absolute method outperforms the other methods at inferring the clustering of distinct groups. Interestingly, this shows that clear cluster- separation is not necessarily correlated with the most precise description of the mutational process. A result with a similar spirit was obtained previously [29] for using MS allele frequency in order to infer the phylogenetic tree of different species/populations. They also found that the best method is not necessarily the one that described the mutational process in the most accurate way.

However, such a measure cannot describe accurately cell depth because depth information is eliminated in the normalization procedure. It is not unreasonable to assume that a Likelihood (or a Bayesian) method tailored towards cell lineage analysis that will make use of all the cells' information, without summarizing it into distance measure, will enable one to infer simultaneously both the topology and the depth in an accurate way. We hope to follow such a path in the future.

We expect that in the coming years next generation sequencing methods will provide us with a much richer genetic signature, and thus improve our ability to infer the cell lineage tree more accurately. This in turn will enable relying on even fine details of the cell lineage tree and not only its rough features.

## Materials and Methods

### Ethics statement

All animal husbandry and euthanasia procedures were performed in accordance with the Institutional Animal Care and Use Committee (IACUC) of the Weizmann Institute of Science.

All human patients signed an informed consent; the study has received Helsinki authorization and was approved by the Rambam Hospital IRB committee and by the Bioethics Committee of the Weizmann Institute of Science.

Our aim is to quantify the performance of different tree reconstruction methods in inferring clustering and depth separation. Most of the methods we tested are distance-based algorithms which use a distance measure between the cells to iteratively join close samples together, such as the Neighbor-Joining algorithm (the full list of methods and distance measures is given below). We tested each method on two features:

1. *Clustering*: The ability to produce distinct clusters for biological groups known to be distinct (e.g., cells from different mice).
2. *Depth separation*: The ability to produce distinct depth differences between cells that are known to have divided a different number of times (e.g., the same cell type from old and young mice).

### Tree reconstruction algorithms

The first distance based algorithm we used is UPGMA (Unweighted Pair Group Method with Arithmetic Mean algorithm) [32] which assumes that all lineages evolve at the same rate. This assumption limits us to reconstructing trees which contain cells that underwent the same (or very similar) number of divisions.

The second algorithm we used is NJ (Neighbor-Joining) [31]. In order to fit the algorithm to our problem, we corrected the branch lengths such that they will not be negative. When negative branches appear during the running of the algorithm, we set its length to 0, adjusting its sibling branch accordingly [40]. Note that this correction does not change the inference of the topology, since it depends only on the distance matrix, and is not affected by the branch lengths of the inferred tree. With the NJ algorithm, a rooted tree can be created by using an out-group, and the root can then effectively be placed on the point in the tree where the edges from the out-group connect. The root in our trees is usually a mix of a wide variety of cell types (a description of the root's determination is given below, in the Data description section).

The third algorithm we used is QMC (Quartet MaxCut) [33], [41]. Quartets-based methods were initially proposed to provide an alternative to maximum likelihood methods, which are computationally intensive. These methods first estimate a set of trees on many four-leaf subsets of the taxa, and then combine them into a tree on the full set of taxa. The QMC method is based on a recursive divide and conquer algorithm that seeks to maximize the ratio between satisfied and violated quartets at each step. The common implementations of the quartet method (including QMC) produce only a tree topology without any explicit information about the branches lengths. Even though it is possible to add depth estimation to the QMC, we limited ourselves to assessing the quality of existing methods without any new features added.

Apart from the distance based algorithms, we tested a Bayesian method for inferring the cell lineage tree. We used the computer software, BATWING [38] which reads in multi-locus haplotype data, a model and prior distribution specifications. This program uses a Markov Chain Monte Carlo (MCMC) method based on coalescent theory to generate approximate random samples from the posterior distributions of parameters such as mutation rates, effective population sizes and growth rates, and times of population splitting events. Even though there is currently no Likelihood or Bayesian tree reconstructing algorithm that uses microsatellites, which was developed to include the unique features of cell lineages, the growing population implementation of BATWING seemed most suited for our study. The priors we used are 1/100 for the mutation rate, and uniform distributions for the effective population size and the population growth rate per generation (on the intervals [10,000 10,000,000] and [0 2] respectively).

### Distance matrices

The distance-based methods require a distance measure between cells, which ideally should be linear with the actual number of divisions separating any two samples, and should provide the most robust tree reconstruction. We have tested several different distance functions. In these functions 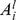 and 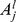 are the number of repeats in the 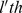 single allele of the 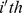 and 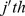 sampled cells, respectively, and 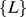 is the set of 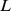 alleles which were amplified for both samples 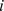 and 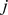 (for autosomal loci, both alleles were included, and for chromosome X loci, one allele was included in male samples):

1. Absolute distance – the distance between two samples, 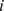 and 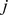, is the average absolute differences between their number of repeats in all alleles which were analyzed in both samples:
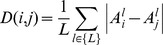

2. Normalized Absolute distance – the distance between two samples, *_i_* and *j*, is the average normalized absolute difference between their number of repeats in all alleles which were analyzed in both samples:
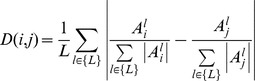
Since normalizing the values eliminates the depth information, we used the depth values from the Absolute distance when performing tree reconstruction using this measure. In order to incorporate the depth 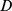 into the reconstructed tree, we used a bottom-up method, where for each pair of sibling leaves we first set their depth according to 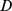 and then calculated the distance of their parent from the root using 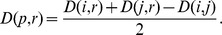 where 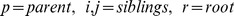. We then continued until reaching the root.
3. Euclidean distance – the distance between two samples, i and j, is:
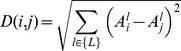

4. “Equal or not” distance – the distance between two samples, 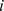 and 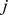, is the number of alleles that differ between the two identifiers:
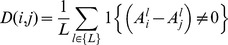

5. Maximum Likelihood (ML) – each entry in the distance matrix is taken as the maximum likelihood estimate of the number of divisions separating the two cells (more details in [7]). The ML estimator is calculated according to two different assumptions: Symmetric Stepwise Mutation Model (SMM) [42] , which assumes an equal probability of addition or deletion of one repeat given that a mutation happens, and a Multistep Mutation Model (MMM) in which multiple additions or deletions of the repeat unit are possible according to a symmetrical distribution. The mutation rate for these step models was set differently for mice and humans. For humans we used the often quoted mutation rate of 1/1000 per meiosis [43]. For mice we used three types of mutation rates: 1. an equal mutation rate for all loci (estimated to be 1/30 from *ex-vivo trees* [7]), 2. different mutation rates according to loci basic unit length (in our panel we have only mono-nucleotide and di-nucleotide repeat loci and their rates were estimated to be 1/22 and 1/32, respectively), and 3. a variable mutation rate linearly dependent on the MS repeat number (with slope and intersection of 0.0183 and 1/2000 respectively, estimated from *ex-vivo* trees).

### Clustering measures

We used three different measures to quantify the quality of the clustering separation ability:

1. Quality of the Largest Cluster (QLC) – this measure focuses on the existence of one large cluster and ignores the behavior of the rest of the cells, and it is calculated as follows. For each cell type 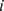, we run over all the internal nodes and count the number of leaves of the 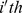 type that are descendants of this node. The degree of the node is defined as 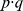, where 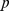 is the percentage of cells from the 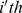 type that are descendants of this node out of all the cells of type 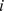, and 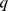 is the percentage of cells from type 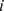 that are descendants of this node among all the cells that are descendants of this node. If 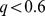, the degree of this node is defined as zero. The score of each cell type 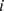 is defined as:
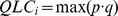
and the 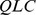 of the whole tree is defined as the average 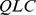 on all the cell types:
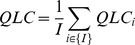
where 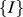 is the set of 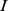 cell types.
2. Tree Entropy (TE) – this measure assesses the amount of mixing on the tree between each pair of cell groups. It is affected only by the number of clusters on the tree that each cell group has, regardless of the sizes of these clusters. The entropy of the whole tree is the average over the entropy matrix containing the entropy between each pair. It is calculated as follows: the number of clusters of each pair of cell types 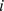 and 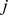 is obtained (here cluster is defined only if all the descendants of a node are of a specific type. Obviously by this definition every leaf is a cluster of size one, unless it is part of a larger cluster). The equivalent state of the system is all the cases that will have the same number of clusters of each type. The number of equivalent states is:
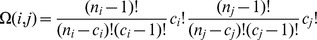
where 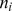 and 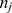 are the number of cells of type 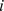 and 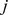, and 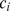 and 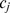 are the number of clusters of type 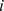 and 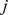. The entropy is:
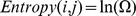
Thus the entropy of the tree is a matrix (half diagonal) and not a scalar. The scalar entropy of the tree is the average over all the pairs 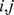:
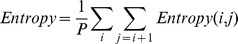
where 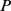 is the number of all pairs 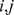.The matrix entropy enables one to analyze all the relationships between the different cell types, and to see which pair is mixed and which pair is separated. As the value of the TE is lower, the various groups of cells in the lineage tree are more clustered, however in order for the figures of this work to be clearer, the TE values presented in them were transformed such that higher values mean better performance (i.e. better clustering).The transformation is: 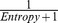

3. Hypergeometric score (HS) – this measure detects a statistically significant clustering of a predefined group of cells on the reconstructed lineage tree. According to the method, given a dichotomous classification of 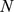 cells in an experiment where 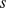 cells belong to group 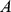 and 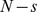 cells belong to the complementary group 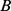, for every branch/internal node in the inferred lineage tree, the null hypotheses of no association between the sub-tree and the classification is tested. This is done by performing a hypergeometric test. Given a sub-tree of 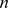 cells in which 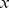 cells are of type 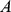 ,the branch's p-value is the probability to see 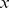 or more cells of type 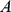 given that the 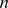 cells are random samples from 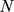:
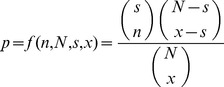
We use a False Discovery Rate correction with an FDR of 20% to determine the p-value threshold for the tree. The Hypergeometric score of the whole tree is defined as:
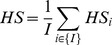
where 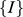 is the set of 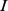 cell types, and 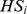 is the most significant p-value of cell type 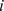.
  The HS values presented in the figures of this work were taken as the log HS values. Since they range from 0 to 1 and are normalized by the best value, the higher normalized values mean better performance (i.e. better clustering).

All these measures evaluate the quality of the separation of the distinct groups on the tree, but they measure parameters that are slightly different. The QLC focuses on the existence of one large cluster and ignores the behavior of the rest of the cells. The TE on the other hand is determined by the number of distinct clusters of each cell type, and ignores their sizes. Hence, the TE focuses on the global behavior of the tree and not just on one sub-tree. The HS, like the QLC, focuses on the existence of a large cluster, but does not ignore the rest of the cells as it detects a statistically significant clustering of a group of cells on the lineage tree.

### Depth measures

We used three different measures to quantify the quality of the depth separation ability:

1. Kolmogorov Smirnov (KS) test – this measure answers the question of whether two datasets were taken from the same distribution. Even if the datasets have the same average but have very different standard deviations, the KS test will reject the null hypothesis of both having the same distribution. For some features of separation, this test answers the required question, but for other features, other tests are necessary, such as the other two measures presented below.
2. Normalized Distance (ND) – this measure evaluates the normalized distance between the averages of two datasets, defined as:
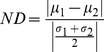
where 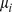 is the average depth of the 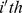 dataset, and 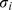 is the standard deviation of the 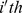 dataset.
3. Overlap percentage – this measure gives the percentage of overlap between two datasets and is defined as follows. For each data point of distribution 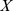, its degree of overlap is the number of data points from distribution 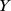 it penetrates. The overlap percentage is defined as:
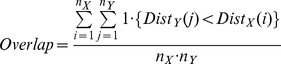

### Data description

#### Mice

Mlh1+/−C57Bl/6 (obtained from Prof. Michael Liskay) [44] and Mlh1+/−129SvEv (provided by Prof. Ari Elson from the Weizmann Institute, Israel) were mated to yield Mlh1−/− progeny of the dual backgrounds. These were used for experiments. All animal husbandry and euthanasia procedures were performed in accordance with the Institutional Animal Care and Use Committee at the Weizmann Institute of Science.

#### Humans

Peripheral Blood (PB) and Bone Marrow (BM) biopsies at diagnosis and relapse collected from leukemia patients were received from the Rambam Healthcare Campus, Haifa, Israel. All patients signed an informed consent, and the study was approved by the RambamIRB committee.

#### Obtaining genetic signature of single cells

The system takes as input a set of DNA samples from individual cells and PCR primers for Microsatellites (MSs), and outputs a reconstructed cell lineage tree of the cells from mutations identified in these MSs. This tree provides an inferred topology and a depth estimation representing the inferred number of cell divisions that occurred along each edge of the lineage tree. The system utilizes a programmable laboratory robot augmented with a PCR and capillary electrophoresis machines. The capillary machine histograms are analyzed by a computer program that utilizes a signal processing algorithm to assign each sample a vector, which is a mathematical representation of the mutations the cell acquired in the MS set. A computer program applies a phylogenetic algorithm to the set of vectors and produces a reconstructed cell lineage tree associated with the DNA samples.

#### Tree reconstruction

Different cell types were sampled from mice and humans (Table S1). The cells were serially diluted in order to apply whole genome amplification (WGA) to individual cells. The genomic DNA obtained was amplified by ∼120 PCR reactions of mouse or human MSs combined in multiplex groups (Tables S2.1 and S2.2). The amplified products were combined and analyzed by capillary electrophoresis followed by computer-aided signal analysis. The size of each locus was determined, thereby providing a genomic signature – the deviation from the putative zygote in the number of MS repeats at each locus (Tables S31, S3.2., S4.1 and S4.2). For mice, the genomic signature of the putative zygote was taken from the tail's genomic DNA of each mouse. The tail contains cells that originated from the ectoderm, endoderm and mesoderm and therefore its genomic signature represents the zygote, or one of its immediate descendants. For humans, the putative zygote signature was taken as the median of the locus size values of all cells. When a tree of several mice or humans was reconstructed, the root signature was a weighted mean of the organisms' putative zygotes.

It is important to note that since all the loci are fully linked, the question of which of the two alleles mutated is not meaningful, as we treat the same locus on the two alleles as two independent loci. This is acceptable because we generally analyzed loci from the chromosome X of males, where there is only one allele. In the few cases where we analyzed loci from other chromosomes (or from chromosome X of a female), we used only loci with very distinct numbers of repeats on each allele, that enable us to identify which of the two alleles underwent mutation, even though we cannot say which copy of the chromosome has which allele.

We should note that aneuploidy, as well as copy number variations (CNV), both of which are frequent in cancer cells, may lead to a situation where even in the male X-chromosome there will be more than one allele. However, in cases with more than one allele the locus was not taken into account for this specific cell. In other chromosomes, the CNV may lead to deletion of one of the alleles which dramatically affects the results, and therefore such loci were not taken into account. Our principle was to analyze only loci in which we detected the amount of alleles expected.

#### Cell isolation

Mice were sacrificed before tissues isolation. Cells were isolated by tissues digestion followed by MACS or FACS. Aliquots of 0.5 µl were spread on a flat bottom 96 well plate and observed under the microscope. Drops that contained single cell were collected into 0.2 ml tubes and subjected to whole genome amplification. Alternatively, cells were isolated by laser micro-dissection as previously demonstrated [45].

#### Whole Genome amplification (WGA) of single cells

WGA was performed using the IllustraGenomiPhi V2 DNA Amplification kit (GE Healthcare Life Sciences, Piscataway, NJ, USA) according to the manufacturer's instructions as described by G. Kumar et al [46]. Briefly, single cells from a 96-well (flat bottom) plate were transferred to PCR tubes (0.2 ml volume) using 3 µlSamplebuffer from the kit. In the optimized protocol, 1.5 µl cell lysissolution (600 mMKOH, 10 mMEDTA, 100 mMdithiothreitol (DTT)) was added to each tube. Cell lysis was carried out for 10 min at 30°C, followed by the addition of 1.5 µl neutralizing solution (4 vol 1 M Tris-HCl, pH 8.0, added to 1 vol of 3 M HCl). WGA reaction was initiated by the addition of a mix composed of 4 µlSample buffer, 9 µlReaction buffer, and 1 µl enzyme, all supplied with the kit. The amplification was then carried out at 30°C for 4 h followed by heat inactivation at 65°C for 10 min.

### Tree simulations

We simulated trees similar to the ones reconstructed using the real data, with some trees containing 3 individuals with 5 different cell types for each individual, and other trees composed of a single individual. We simulated several kinds of topologies, which were different from each other in branch length. For example, in one topology the distance between the leaves and their MRCA was high, compared to the distance between the root and these MRCAs, and in another topology this distance between the leaves and their MRCA was much lower. We repeated the simulation 1000 times, where in each iteration, we built a random tree and randomly added MS mutations according to a given mutation rate (1/100, 1/1000 and 1/10000) using a binomial distribution. We then reconstructed the tree using all of our methods and compared it with the actual tree that was generated. The topology comparison between the inferred and the actual trees was done using Penny & Hendy's topological distance algorithm [47]. In this algorithm each internal edge confers a partitioning of the tree into two groups by removing the edge. We assigned a score equal to the ratio of equal partitions of the two trees to the total number of partitions. For each of the simulated trees we also calculated the clustering measures (mentioned above).

## Supporting Information

Figure S1 **Schematic description of the lineage relationship between cells from two brothers.** Blue circles represent zygotes (the two lower ones are the zygotes of two brothers, and the upper one is the zygote of their mother), red circles represent cells from the brothers, which were harvested at the same time point. Stage 1 starts at the mother's zygote and ends at the zygotes of the brothers. Stage 2 starts at the zygotes of the brothers and ends at the time the cells were harvested.
(TIF)

Figure S2 **The percentage of separation between the two mice as a function of the ratio between the two stages.** The number of cells we used for each mice were 3 (black), 10 (blue), and 50 (red). The rest of the parameters are: 50 loci, a mutation rate of 1 mutation per 100 divisions and stage 1 is 40 divisions. The X axis is the ratio between stage 2 to stage 1, and the Y axis is the percentage of fully separated mice. It can be seen that below ratio 2, the percentage is almost 1, and above ratio 2, the percentage declines sharply.
(TIF)

Figure S3 **The percentage of separation between the two mice as a function of the number of loci.** The mutation rates used are 0.1 (blue), 0.001 (red), 0.0005 (turquoise), and 0.0001 (black). The ratio between stage 1 and stage 2 is 5. It can be seen that as the mutation rate gets higher, less loci are needed in order to obtain a separation of above 90%.
(TIF)

Figure S4 **Lineage trees of one dataset of cells from three individuals: M1 (turquoise), M2 (red) and M6 (green).** All the trees were reconstructed using the NJ algorithm with the following distance matrices: (A) Absolute (B) Normalized- Absolute (C) Equal or Not (D) Euclidean (E) SMM with equal mutation rates (F) SMM with a different mutation rate for mono repeats and a different mutation rate for di repeats (G) SMM with length dependent mutation rates (H) MMM with equal mutation rates (I) MMM with a different mutation rate for mono repeats and a different mutation rate for di repeats (J) MMM with length dependent mutation rates.
(TIF)

Figure S5 **Lineage trees of one dataset of cells from a single individual (M1).** Each cell type is colored by a different color. All the trees were reconstructed using the NJ algorithm with the following distance matrices: (A) Absolute (B) Normalized- Absolute (C) Equal or Not (D) Euclidean (E) SMM with equal mutation rates (F) SMM with a different mutation rate for mono repeats and a different mutation rate for di repeats (G) SMM with length dependent mutation rates (H) MMM with equal mutation rates (I) MMM with a different mutation rate for mono repeats and a different mutation rate for di repeats (J) MMM with length dependent mutation rates.
(TIF)

Figure S6 **Lineage trees of one dataset of cells from two individuals. M2 (red) and M3 (blue), composed of one cell type (oocytes).** All the trees were reconstructed using the NJ algorithm with the following distance matrices: (A) Absolute (B) Normalized- Absolute (C) Equal or Not (D) Euclidean (E) SMM with equal mutation rates (F) SMM with a different mutation rate for mono repeats and a different mutation rate for di repeats (G) SMM with length dependent mutation rates (H) MMM with equal mutation rates (I) MMM with a different mutation rate for mono repeats and a different mutation rate for di repeats (J) MMM with length dependent mutation rates.
(TIF)

Figure S7 **Performance summary of all the methods on all the datasets of mice and humans.** Left panel – Mouse, right panel- Human. Each column presents a different clustering measure (see Materials and Methods for details), and each bar represents a different distance measure, where the colors specify the distance measures as noted in the legend. The first group of bars (from left to right) presents the results using the NJ algorithm, the second group of bars presents the results using the QMC algorithm, the third presents the results using the UPGMA algorithm, and the last one presents the results using the BATWING tool. Rows description: (A) The average score of all the methods, where higher values (that are transformations of the real scores) indicate better performance. (B) The normalized average scores in which again, higher values mean better performance. (C) The average rank of each method. (D) The number of times every method received the highest rank (for the mouse panel the highest rank is 31 since we compared 31 methods, and for the human panel the highest rank is 19).
(TIF)

Figure S8 **Examples of simulated trees composed of multiple individuals and a single individual.** Shown are two kinds of topologies of the simulated trees which are different from each other in their branch lengths. (A) The distance between the leaves and their MRCA is high, compared to the distance between the root and these MRCAs, (B) The distance between the leaves and their MRCA is low, compared to the distance between the root and these MRCAs. The left tree in each panel is a simulated tree composed of three individuals and the right tree is a simulated tree composed of one individual.
(TIF)

Figure S9 **Clustering simulation results.** (A) Results of simulated trees composed of multiple individuals. (B) Results of simulated trees composed of one individual. Left panels present the results of the simulation of topology A, and Right panels present the results of the simulation of topology B (shown in Figure S8). Each column presents a different clustering measure (see Materials and Methods for details), and each bar represents a different distance measure, where the colors specify different amounts of loci (50, 100 and 500). Each row presents a different mutation rate (1/100, 1/1000 and 1/10,000). The values are the average score, where higher values (that are transformations of the real scores) indicate better performance.
(TIF)

Figure S10 **Performance summary of all the methods for datasets composed of cells of same depth only.** Each column presents a different clustering measure (see Materials and Methods for details), and each bar represents a different distance measure, where the colors specify the distance measures as noted in the legend. The first group of bars (from left to right) presents the results using the NJ algorithm, the second group of bars presents the results using the QMC algorithm, the third presents the results using the UPGMA algorithm, and the last one presents the results using the BATWING tool. Rows description: (A) The average score of all the methods, where higher values (that are transformations of the real scores) indicate better performance. (B) The normalized average scores in which again, higher values mean better performance. (C) The average rank of each method. (D) The number of times every method received the highest rank (in this case it is 31 since we compared 31 methods).
(TIF)

Figure S11 **Performance summary of all the methods for datasets composed of cells from single individuals.** Left panel - Mouse, right panel - Human. Each column presents a different clustering measure (see Materials and Methods for details), and each bar represents a different distance measure, where the colors specify the distance measures as noted in the legend. The first group of bars (from left to right) presents the results using the NJ algorithm, the second group of bars presents the results using the QMC algorithm, the third presents the results using the UPGMA algorithm, and the last one presents the results using the BATWING tool. Rows description: (A) The average score of all the methods, where higher values (that are transformations of the real scores) indicate better performance. (B) The normalized average scores in which again, higher values mean better performance. (C) The average rank of each method. (D) The number of times every method received the highest rank (for the mouse panel the highest rank is 31 since we compared 31 methods, and for the human panel the highest rank is 19).
(TIF)

Figure S12 **Performance summary of all the methods for datasets composed of one cell type.** Left panel - Mouse, right panel - Human. Each column presents a different clustering measure (see Materials and Methods for details), and each bar represents a different distance measure, where the colors specify the distance measures as noted in the legend. The first group of bars (from left to right) presents the results using the NJ algorithm, the second group of bars presents the results using the QMC algorithm, the third presents the results using the UPGMA algorithm, and the last one presents the results using the BATWING tool. Rows description: (A) The average score of all the methods, where higher values (that are transformations of the real scores) indicate better performance. (B) The normalized average scores in which again, higher values mean better performance. (C) The average rank of each method. (D) The number of times every method received the highest rank (for the mouse panel the highest rank is 31 since we compared 31 methods, and for the human panel the highest rank is 19).
(TIF)

Figure S13 **Depth quality of the different methods.** Each column presents a different depth quality measure (see Materials and Methods for details), and each bar represents a different distance measure, where the colors specify the distance measures as noted in the legend. Rows description: (A) The average depth score of all the methods, where lower values for the KS test, lower values for the overlap percentage and higher values for the normalized distance test mean better performance. (B) The normalized average scores in which again, higher values mean better performance. (C) The average rank of each method. (D) The number of times every method received the highest rank (in this case it is 10 since we compared 10 methods).
(TIF)

Figure S14 **Depth simulation results.** Left panels present the results of the simulation of topology A where the cells are shallow, and Right panels present the results of the simulation of topology B where the cells are deep. Each column presents a different depth measure (see Materials and Methods for details), and each bar represents a different distance measure, where the colors specify different amounts of loci (50, 100 and 500). Each row presents a different mutation rate (1/100, 1/1000 and 1/10,000). The values are the average score, where higher values of KS and Normalized-Distance, and lower values of the Overlap percentage indicate better performance.
(TIF)

Table S1 **Description of the organisms we used for our research and cell types which were isolated from them.** The description contains the name, species, gender, age of the organism, and the cell types which were extracted from it.
(XLSX)

Table S2 **Mouse (S2.1) and Human (S2.2) microsatellite panel.** Description of the microsatellite loci we used in our experiments. The tables contain the loci names, their size and their forward and reverse primers. Loci name (first column): [Organism type][Chromosome]_[Basic unit][number of repeats]_[serial number]. Organism type can be M for mouse or H for human, Chromosome can be a number or the letter X.
(XLSX)

Table S3 **Fragment sizes of Mouse (S3.1) and Human (S3.2).** The tables contain information of each cell we have analyzed. This includes the sample's name (Sample ID), the name of the animal it was taken from (Animal ID) and a description of its origin (Tissue). In addition it contains the fragment sizes which were calculated from the capillary machine histograms. These sizes include the locus length and the primers which were designed to amplify this locus. Each two following columns present the sizes of 2 alleles in the same locus. In the header line, the names of the loci are presented. ‘X’ represents missing data. Empty cells indicate that this current locus was not measured for this sample.
(XLSX)

Table S4 **Absolute loci lengths of Mouse (S4.1) and Human (S4.2).** The tables contain information of each cell we have analyzed. This includes the sample's name (Sample ID), the name of the animal it was taken from (Animal ID) and a description of its origin (Tissue). In addition it contains the absolute loci lengths which were evaluated as the fragment sizes (calculated from the capillary machine histograms) minus the primers lengths. Each two following columns present the sizes of 2 alleles in the same locus. In the header line, the names of the loci are presented. ‘X’ represents missing data. Empty cells indicate that this current locus was not measured for this sample.
(XLSX)

Table S5 **Results** **of clustering and depths tests.** Clustering: tables S5.1 (all datasets of Mouse), S5.2 (all datasets of Human) and S5.3 (datasets with the same cell depth) present the results of the three clustering measures (QLC, TE and HS) over 31 reconstructed algorithms (10 NJ methods, 10 QMC methods, 10 UPGMA methods and the Batwing method). When the value of the measure QLC is higher the performance is better, whereas the opposite occurs for the measures TE and HS where a lower value means better performance. Depths: table S5.4 presents the results of the three depth measures (KS, Norm-D and Overlap). When the values of the measures KS and Overlap are lower the performance is better, whereas the opposite occurs for the measure Norm-D in which a higher value means better performance. The results are presented over 10 NJ reconstructed algorithms. In all tables, first column - the dataset name [List of organisms ID's]_[cell type]. If the cell type is ‘all’ it means that all the cells in the organisms were used. If the list of organisms contains only one organism, it means that the clustering test was performed over the different cell types of the organism.
(XLSX)

Table S6 **Topology comparison simulation results of multiple individuals (S6.1) and single individuals (S6.2).** The tables present the results of the comparison between topologies of the inferred trees and the simulated trees, which was done using Penny & Hendy's topological distance algorithm. The top part of the table refers to the simulated trees of topology A, and the other part of the table refers to the simulated trees of topology B (both topologies are presented in Figure S8). For each topology, the simulations performed over three mutation rates (1/100, 1/1000, and 1/10000) and over three amounts of loci (50, 100 and 500). The values presented in the table are the average scores of 1000 simulations. The highest value which can be achieved is 1 (Higher values are better) and it can be seen that none of the methods in any of the scenarios is close to that value.
(XLSX)

Table S7 **Clustering simulation results of multiple individuals (S7.1) and single individuals (S7.2).** The tables present the clustering measures of the lineage tree reconstruction of the simulated trees (described in Methods and Materials). The top part of the table refers to the simulated trees of topology A, and the other part of the table refers to the simulated trees of topology B (both topologies are presented in Figure S8). For each topology, the simulations performed over three mutation rates (1/100, 1/1000, and 1/10000) and over three amounts of loci (50, 100 and 500). The values presented in the table are the average scores of 1000 simulations. Higher values of QLC and lower values of TE and HS are better. The different clustering measures are described in Methods and Materials.
(XLSX)

Table S8 **Depth simulation results.** The table presents the results of the depth simulations. The top part of the table refers to simulated trees with relatively low depths of cells (20 and 50 divisions), and the other part of the table refers to the simulated trees with relatively high depths of cells (250 and 300 divisions). For each scenario, the simulations were performed over three mutation rates (1/100, 1/1000, and 1/10000) and over three amounts of loci (50, 100 and 500). The values are the average results of 1000 simulations. The bold values are the best ones over the different distance matrices (lower values of KS and Overlap and higher values of Norm-D are better).The different depth measures are described in Methods and Materials.
(XLSX)

Table S9 **Reliability of reconstructed lineage trees.** #1–#8 are datasets on which the clustering separation was checked, #9–#11 are datasets on which the depths separation was checked. The bootstrap scores are the average scores over the sampled trees. The TE score was normalized by dividing it with the score of a tree with random permutations of the leaves. For details about the different scores see Materials and Methods.
(XLSX)

Text S1 **Computer simulations for separation analysis between related mice.**
(DOC)
