# Supplementary figures and images for: Comparing Algorithms That Reconstruct Cell Lineage Trees Utilizing Information on Microsatellite Mutations
Source: PLoS Comput Biol. 2013 Nov 14;9(11):e1003297. doi: 10.1371/journal.pcbi.1003297 (PMC3828138; doi:10.1371/journal.pcbi.1003297)

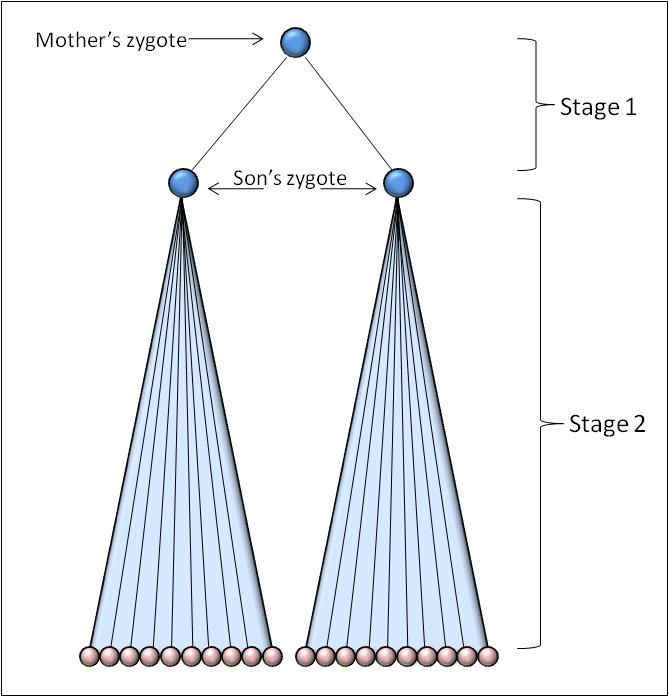

Supplement: Figure S1 — Schematic description of the lineage relationship between cells from two brothers. Blue circles represent zygotes (the two lower ones are the zygotes of two brothers, and the upper one is the zygote of their mother), red circles represent cells from the brothers, which were harvested at the same time point. Stage 1 starts at the mother's zygote and ends at the zygotes of the brothers. Stage 2 starts at the zygotes of the brothers and ends at the time the cells were harvested. (TIF) [file pcbi.1003297.s001.tif]

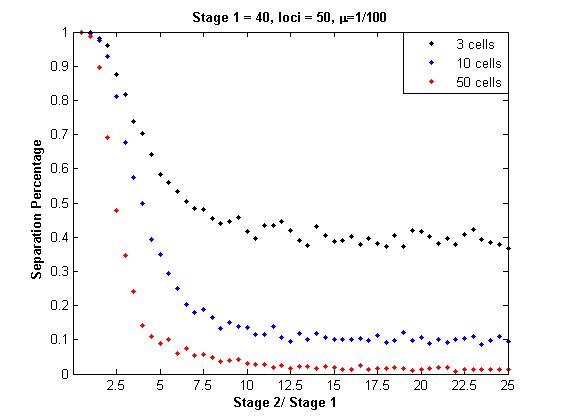

Supplement: Figure S2 — The percentage of separation between the two mice as a function of the ratio between the two stages. The number of cells we used for each mice were 3 (black), 10 (blue), and 50 (red). The rest of the parameters are: 50 loci, a mutation rate of 1 mutation per 100 divisions and stage 1 is 40 divisions. The X axis is the ratio between stage 2 to stage 1, and the Y axis is the percentage of fully separated mice. It can be seen that below ratio 2, the percentage is almost 1, and above ratio 2, the percentage declines sharply. (TIF) [file pcbi.1003297.s002.tif]

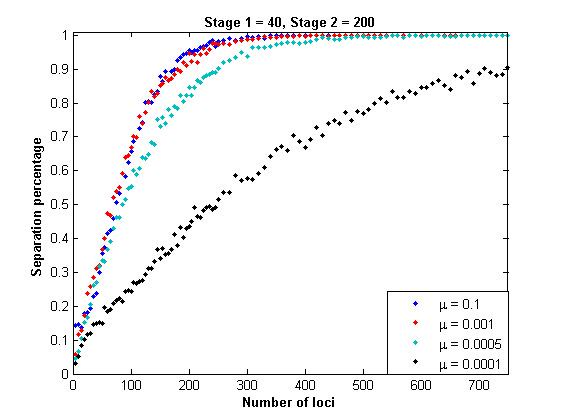

Supplement: Figure S3 — The percentage of separation between the two mice as a function of the number of loci. The mutation rates used are 0.1 (blue), 0.001 (red), 0.0005 (turquoise), and 0.0001 (black). The ratio between stage 1 and stage 2 is 5. It can be seen that as the mutation rate gets higher, less loci are needed in order to obtain a separation of above 90%. (TIF) [file pcbi.1003297.s003.tif]

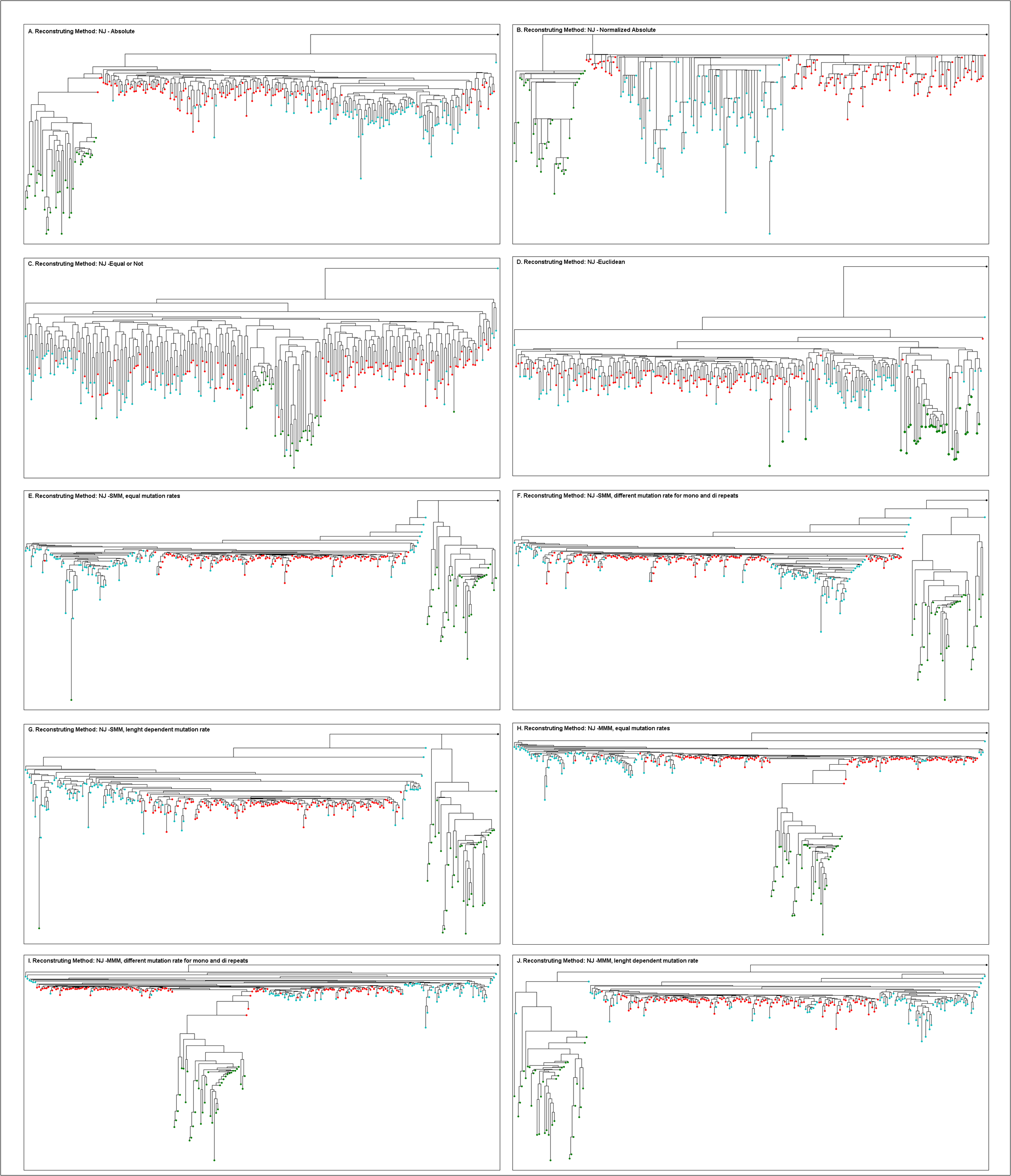

Supplement: Figure S4 — Lineage trees of one dataset of cells from three individuals: M1 (turquoise), M2 (red) and M6 (green). All the trees were reconstructed using the NJ algorithm with the following distance matrices: (A) Absolute (B) Normalized- Absolute (C) Equal or Not (D) Euclidean (E) SMM with equal mutation rates (F) SMM with a different mutation rate for mono repeats and a different mutation rate for di repeats (G) SMM with length dependent mutation rates (H) MMM with equal mutation rates (I) MMM with a different mutation rate for mono repeats and a different mutation rate for di repeats (J) MMM with length dependent mutation rates. (TIF) [file pcbi.1003297.s004.tif]

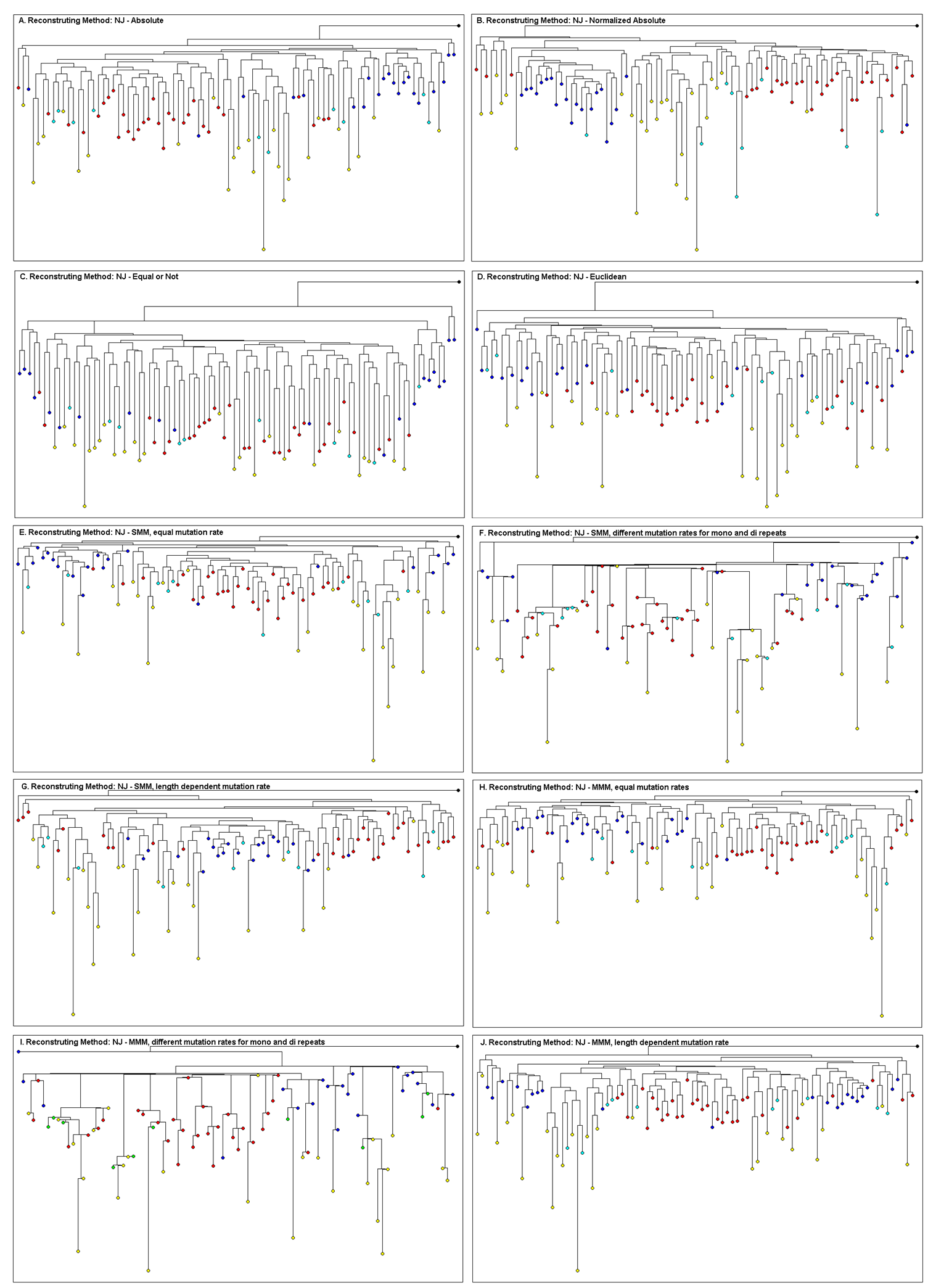

Supplement: Figure S5 — Lineage trees of one dataset of cells from a single individual (M1). Each cell type is colored by a different color. All the trees were reconstructed using the NJ algorithm with the following distance matrices: (A) Absolute (B) Normalized- Absolute (C) Equal or Not (D) Euclidean (E) SMM with equal mutation rates (F) SMM with a different mutation rate for mono repeats and a different mutation rate for di repeats (G) SMM with length dependent mutation rates (H) MMM with equal mutation rates (I) MMM with a different mutation rate for mono repeats and a different mutation rate for di repeats (J) MMM with length dependent mutation rates. (TIF) [file pcbi.1003297.s005.tif]

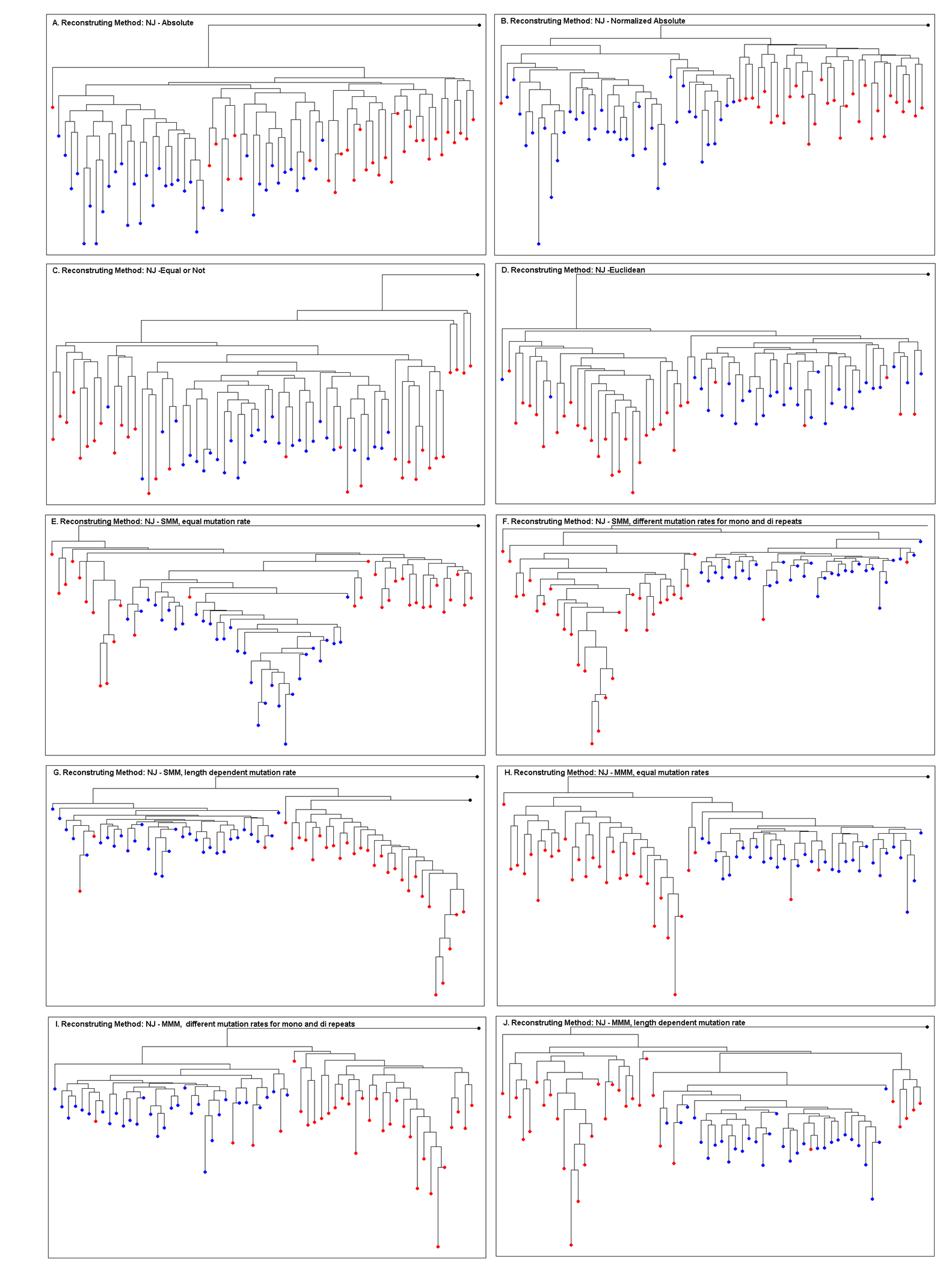

Supplement: Figure S6 — Lineage trees of one dataset of cells from two individuals. M2 (red) and M3 (blue), composed of one cell type (oocytes). All the trees were reconstructed using the NJ algorithm with the following distance matrices: (A) Absolute (B) Normalized- Absolute (C) Equal or Not (D) Euclidean (E) SMM with equal mutation rates (F) SMM with a different mutation rate for mono repeats and a different mutation rate for di repeats (G) SMM with length dependent mutation rates (H) MMM with equal mutation rates (I) MMM with a different mutation rate for mono repeats and a different mutation rate for di repeats (J) MMM with length dependent mutation rates. (TIF) [file pcbi.1003297.s006.tif]

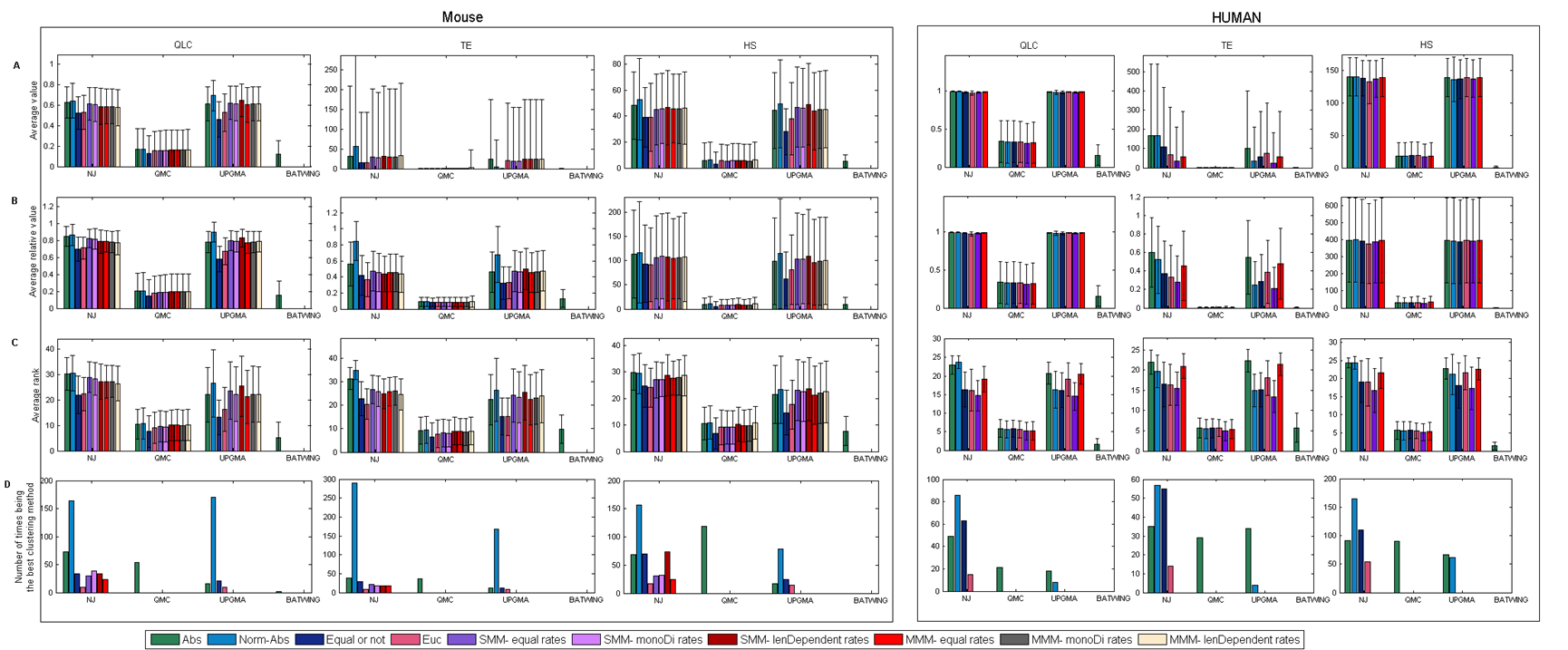

Supplement: Figure S7 — Performance summary of all the methods on all the datasets of mice and humans. Left panel – Mouse, right panel- Human. Each column presents a different clustering measure (see Materials and Methods for details), and each bar represents a different distance measure, where the colors specify the distance measures as noted in the legend. The first group of bars (from left to right) presents the results using the NJ algorithm, the second group of bars presents the results using the QMC algorithm, the third presents the results using the UPGMA algorithm, and the last one presents the results using the BATWING tool. Rows description: (A) The average score of all the methods, where higher values (that are transformations of the real scores) indicate better performance. (B) The normalized average scores in which again, higher values mean better performance. (C) The average rank of each method. (D) The number of times every method received the highest rank (for the mouse panel the highest rank is 31 since we compared 31 methods, and for the human panel the highest rank is 19). (TIF) [file pcbi.1003297.s007.tif]

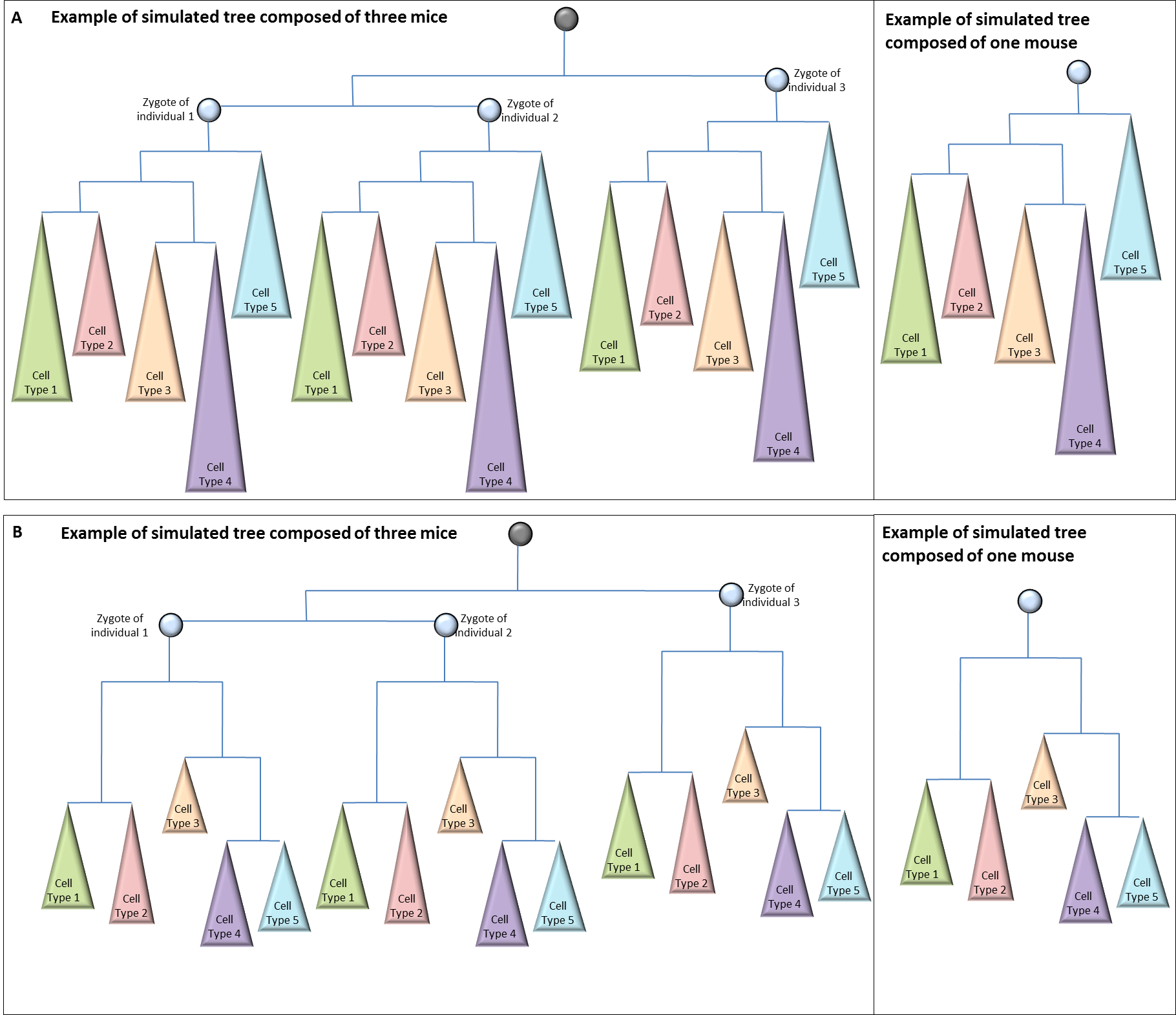

Supplement: Figure S8 — Examples of simulated trees composed of multiple individuals and a single individual. Shown are two kinds of topologies of the simulated trees which are different from each other in their branch lengths. (A) The distance between the leaves and their MRCA is high, compared to the distance between the root and these MRCAs, (B) The distance between the leaves and their MRCA is low, compared to the distance between the root and these MRCAs. The left tree in each panel is a simulated tree composed of three individuals and the right tree is a simulated tree composed of one individual. (TIF) [file pcbi.1003297.s008.tif]

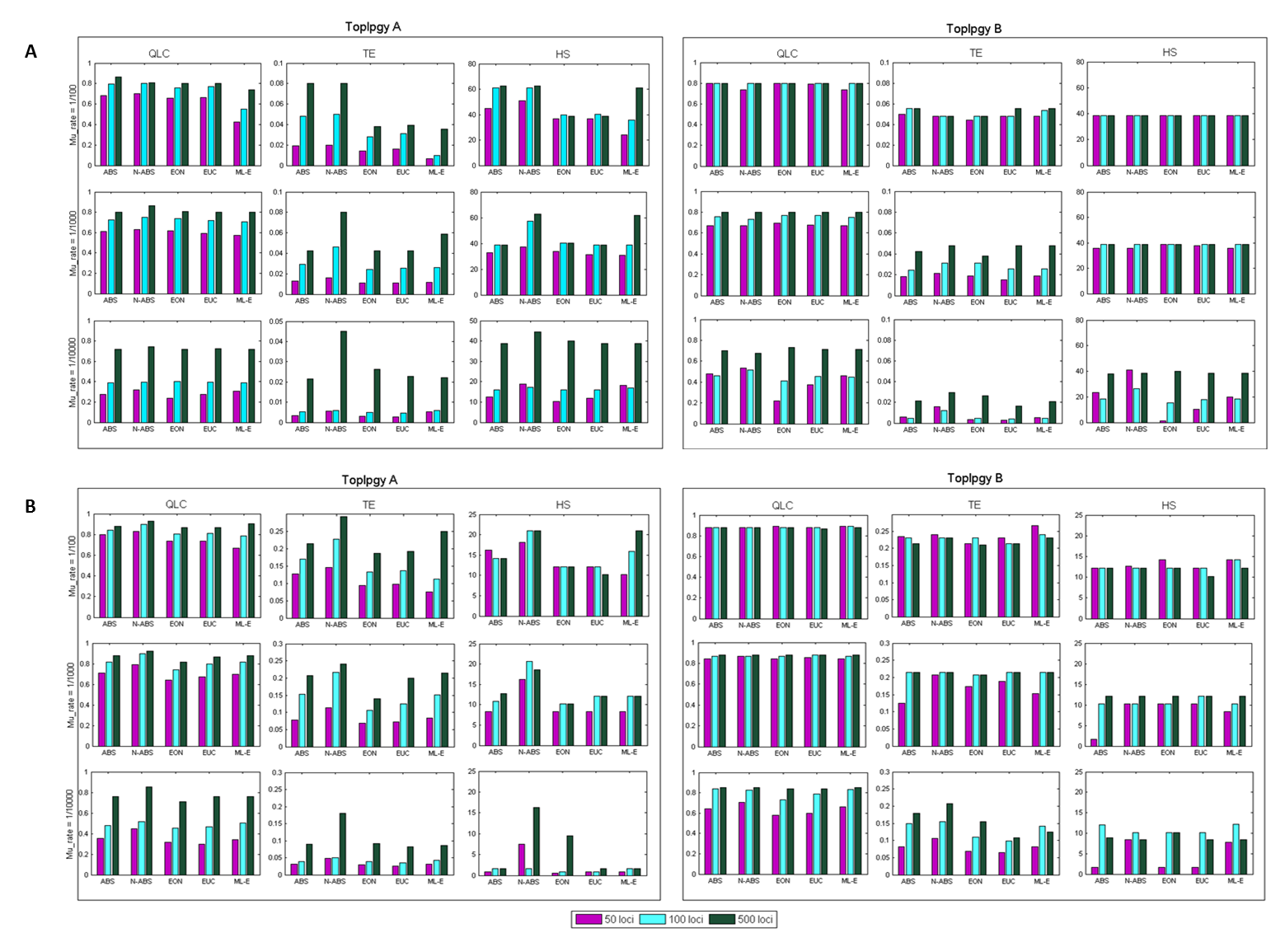

Supplement: Figure S9 — Clustering simulation results. (A) Results of simulated trees composed of multiple individuals. (B) Results of simulated trees composed of one individual. Left panels present the results of the simulation of topology A, and Right panels present the results of the simulation of topology B (shown in Figure S8). Each column presents a different clustering measure (see Materials and Methods for details), and each bar represents a different distance measure, where the colors specify different amounts of loci (50, 100 and 500). Each row presents a different mutation rate (1/100, 1/1000 and 1/10,000). The values are the average score, where higher values (that are transformations of the real scores) indicate better performance. (TIF) [file pcbi.1003297.s009.tif]

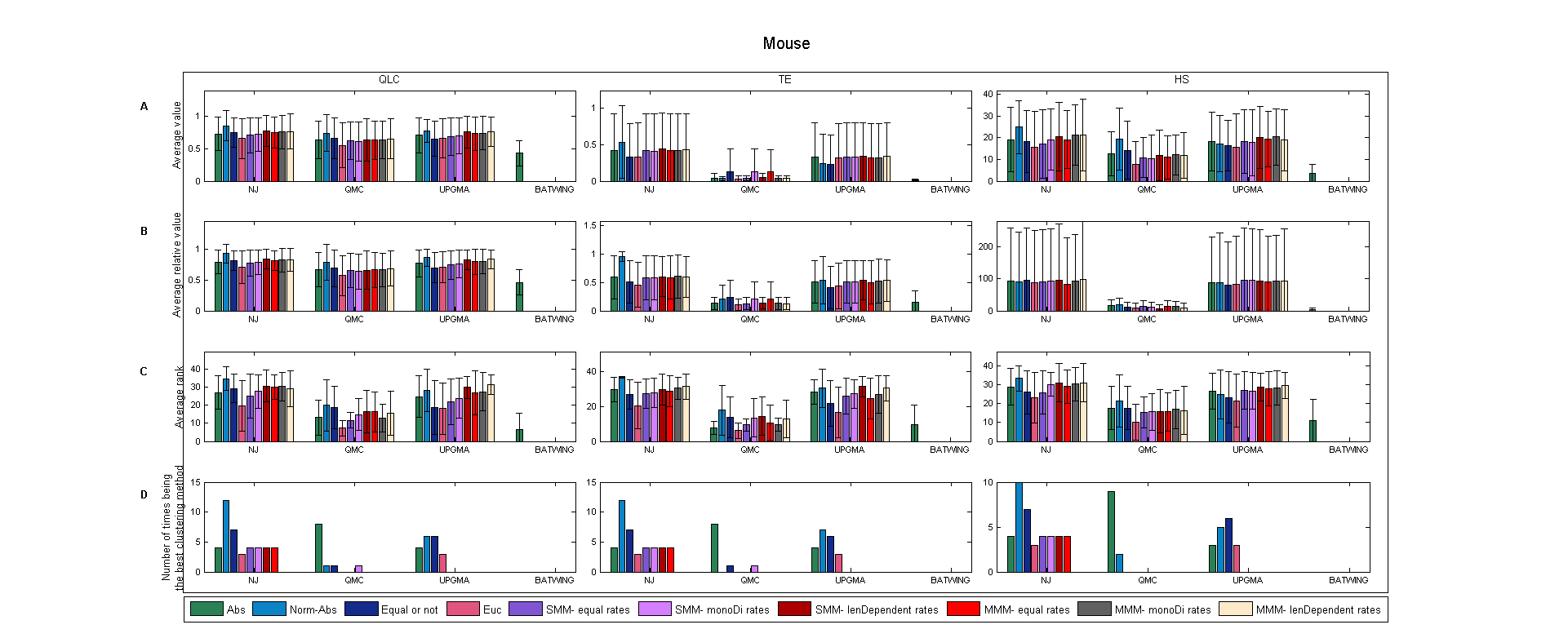

Supplement: Figure S10 — Performance summary of all the methods for datasets composed of cells of same depth only. Each column presents a different clustering measure (see Materials and Methods for details), and each bar represents a different distance measure, where the colors specify the distance measures as noted in the legend. The first group of bars (from left to right) presents the results using the NJ algorithm, the second group of bars presents the results using the QMC algorithm, the third presents the results using the UPGMA algorithm, and the last one presents the results using the BATWING tool. Rows description: (A) The average score of all the methods, where higher values (that are transformations of the real scores) indicate better performance. (B) The normalized average scores in which again, higher values mean better performance. (C) The average rank of each method. (D) The number of times every method received the highest rank (in this case it is 31 since we compared 31 methods). (TIF) [file pcbi.1003297.s010.tif]

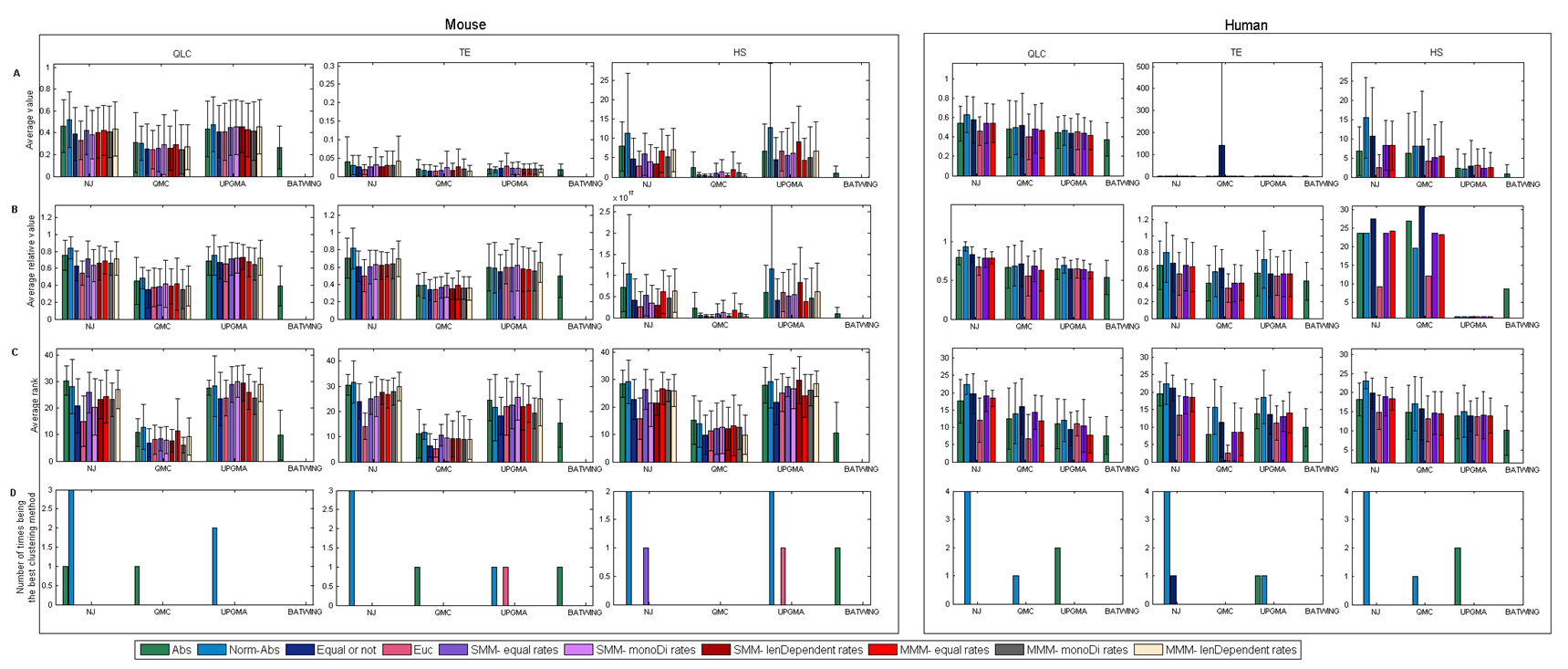

Supplement: Figure S11 — Performance summary of all the methods for datasets composed of cells from single individuals. Left panel - Mouse, right panel - Human. Each column presents a different clustering measure (see Materials and Methods for details), and each bar represents a different distance measure, where the colors specify the distance measures as noted in the legend. The first group of bars (from left to right) presents the results using the NJ algorithm, the second group of bars presents the results using the QMC algorithm, the third presents the results using the UPGMA algorithm, and the last one presents the results using the BATWING tool. Rows description: (A) The average score of all the methods, where higher values (that are transformations of the real scores) indicate better performance. (B) The normalized average scores in which again, higher values mean better performance. (C) The average rank of each method. (D) The number of times every method received the highest rank (for the mouse panel the highest rank is 31 since we compared 31 methods, and for the human panel the highest rank is 19). (TIF) [file pcbi.1003297.s011.tif]

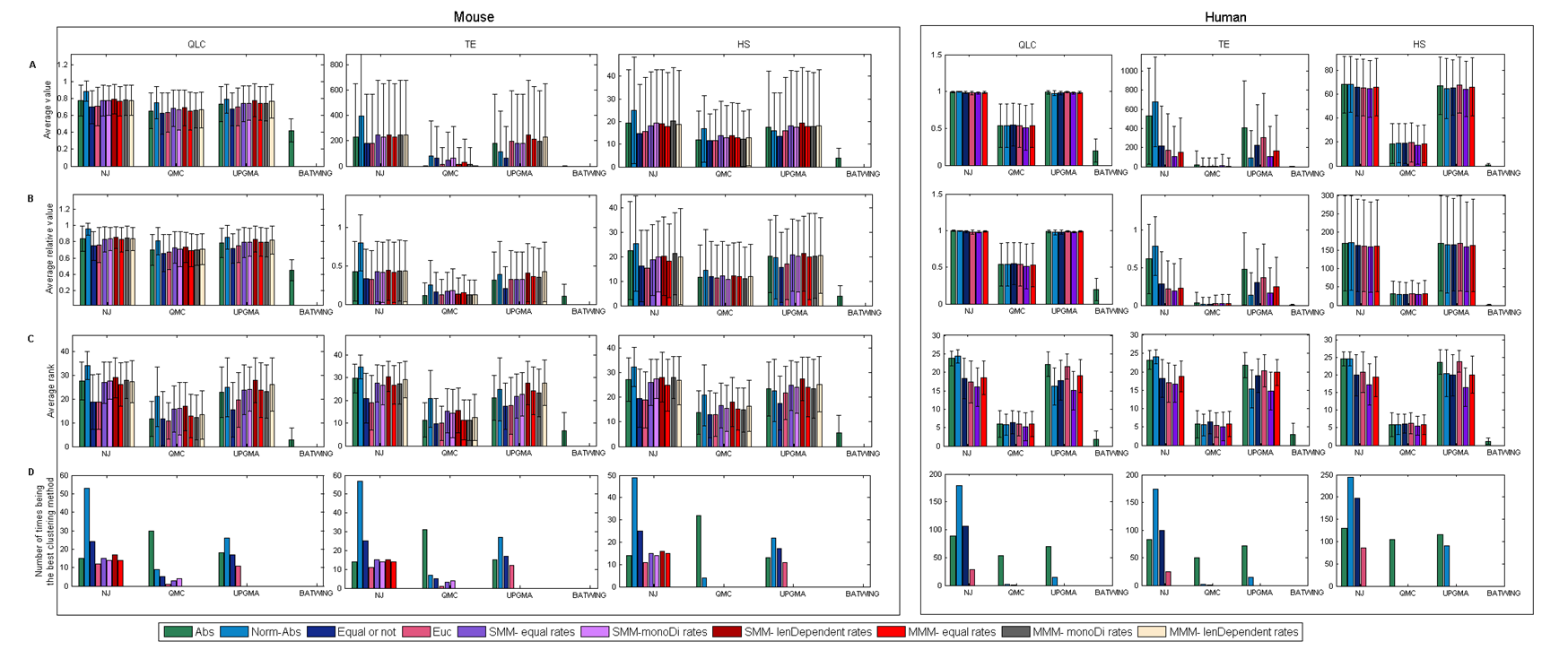

Supplement: Figure S12 — Performance summary of all the methods for datasets composed of one cell type. Left panel - Mouse, right panel - Human. Each column presents a different clustering measure (see Materials and Methods for details), and each bar represents a different distance measure, where the colors specify the distance measures as noted in the legend. The first group of bars (from left to right) presents the results using the NJ algorithm, the second group of bars presents the results using the QMC algorithm, the third presents the results using the UPGMA algorithm, and the last one presents the results using the BATWING tool. Rows description: (A) The average score of all the methods, where higher values (that are transformations of the real scores) indicate better performance. (B) The normalized average scores in which again, higher values mean better performance. (C) The average rank of each method. (D) The number of times every method received the highest rank (for the mouse panel the highest rank is 31 since we compared 31 methods, and for the human panel the highest rank is 19). (TIF) [file pcbi.1003297.s012.tif]

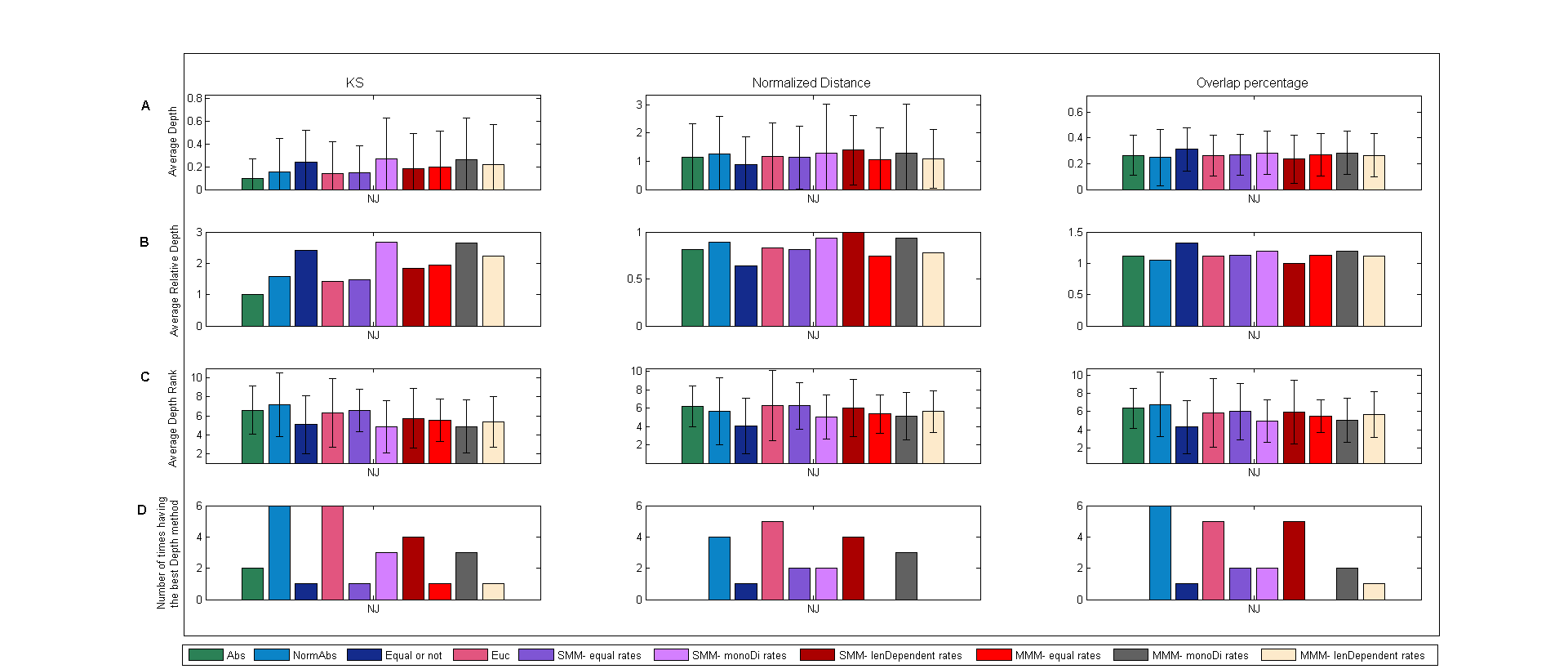

Supplement: Figure S13 — Depth quality of the different methods. Each column presents a different depth quality measure (see Materials and Methods for details), and each bar represents a different distance measure, where the colors specify the distance measures as noted in the legend. Rows description: (A) The average depth score of all the methods, where lower values for the KS test, lower values for the overlap percentage and higher values for the normalized distance test mean better performance. (B) The normalized average scores in which again, higher values mean better performance. (C) The average rank of each method. (D) The number of times every method received the highest rank (in this case it is 10 since we compared 10 methods). (TIF) [file pcbi.1003297.s013.tif]

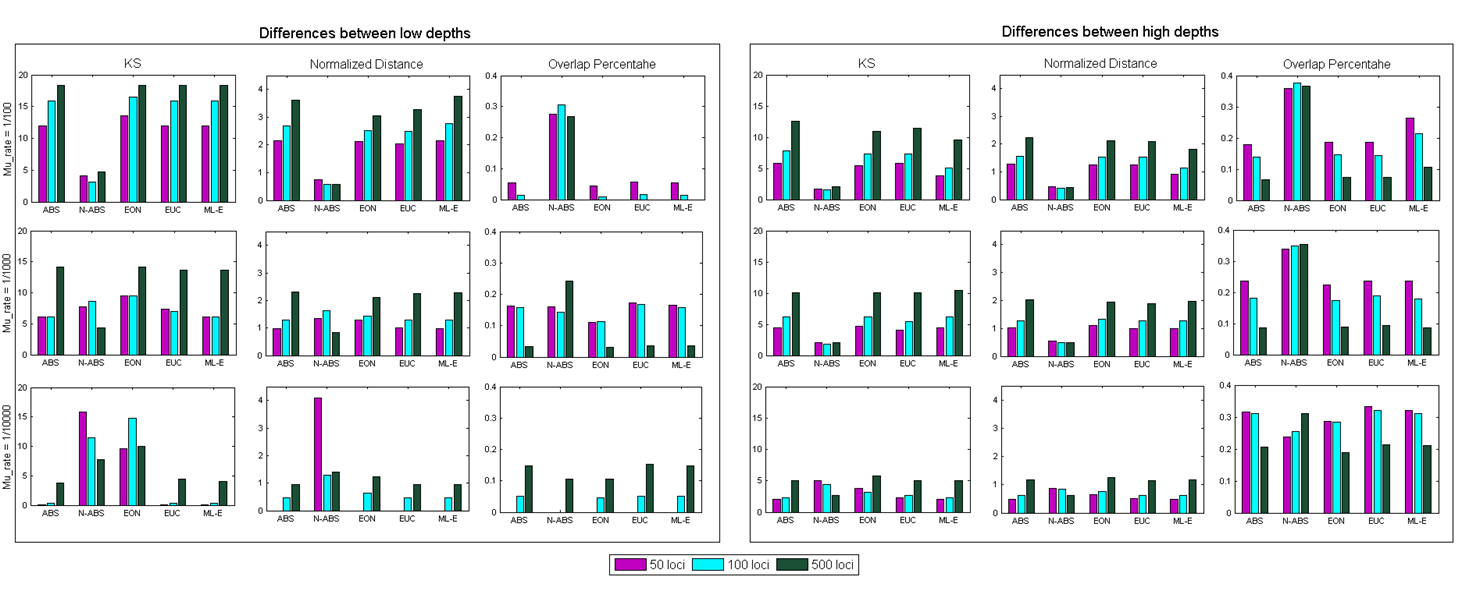

Supplement: Figure S14 — Depth simulation results. Left panels present the results of the simulation of topology A where the cells are shallow, and Right panels present the results of the simulation of topology B where the cells are deep. Each column presents a different depth measure (see Materials and Methods for details), and each bar represents a different distance measure, where the colors specify different amounts of loci (50, 100 and 500). Each row presents a different mutation rate (1/100, 1/1000 and 1/10,000). The values are the average score, where higher values of KS and Normalized-Distance, and lower values of the Overlap percentage indicate better performance. (TIF) [file pcbi.1003297.s014.tif]
