# Supplementary material for: Comparing Algorithms That Reconstruct Cell Lineage Trees Utilizing Information on Microsatellite Mutations
Source: PLoS Comput Biol. 2013 Nov 14;9(11):e1003297. doi: 10.1371/journal.pcbi.1003297 (PMC3828138; doi:10.1371/journal.pcbi.1003297)
Supplement: Text S1 — Computer simulations for separation analysis between related mice. (DOC) [file pcbi.1003297.s024.doc]

# Separation between related mice

For the sake of simplicity we analyzed few cell types, each obtained from two male brothers, however even more complicated family relationships behave according to the same principle. The lineage relations between cells in the two brothers can be divided into two stages; Stage 1, from the zygote of the brothers' mother until the zygotes of the brothers, and Stage 2 from the brothers' zygotes until the time the cells were harvested. Again for simplicity's sake, we will assume that the different cell types split from each other in the very early embryonic stages, an assumption which is not necessarily required. A schematic description of the cell-lineage of the cells in the two brothers is presented in Figure S1.

In distinction from single nucleotide polymorphisms, repeated mutations in MSs cannot be ignored. Due to their high mutation rate and the fact that both insertions and deletions may occur, the information that describes the separation between the two brothers may be erased by new mutations in the second stage.

We tested numerically this effect in the following way. We assumed a model where the true cell lineage of the cells is exactly the two stages model we described above. We assigned mutations for each locus for each branch from a binomial distribution, with the parameters being the branch length and the mutation rate per loci per division. Then, we assigned the genetic signature of each cell by tracking all the mutations it acquired since the zygote until the present, and reconstructed the cell lineage tree using the NJ-Absolute method. Obviously, unless the number of loci is infinite we will probably not reconstruct the real tree and there will be some deviations from it. There will be cases where these deviations will be so large that they may even mix the cells from the mice together.

For different ratios between stage 1 and stage 2, we produced 1000 genetic signatures per ratio, and measured the number of datasets that present a full separation between the two mice. Figure S2 presents the percentage of fully separated mice as a function of the ratio between the two stages. It can be seen that below ratio 2 the percentage of separated datasets is almost 1, and above ratio 2, the percentage declines sharply. The different colors show the separation percentage for different numbers of cells from each mouse. As the number of cells grows, the separation declines. We have found (not shown here) that the separation percentages depend mainly on the ratio between the two stages, and only weakly on the absolute length of the stages.

Another parameter that affects strongly the separation percentage is the number of loci. In Figure S3 we show the separation percentage as a function of the number of loci being used. The different colors show the separation percentage for different mutation rates. It can be seen that for a ratio of 5 between stage 1 and stage 2, as the mutation rate gets higher, less loci are needed in order to obtain a separation of above 90%, and above 750 loci using all the mutation rates we obtain a 90% separation.

Our data does not contain a fixed number of loci per cell due to the fact that the PCR process does not amplify the DNA equally in all cases. On the average, we have about 80 loci per cell, but for the distance calculation between two cells, we allowed the use of fewer loci, with the limitation of having more than 25 common loci.

The meaning of these results obtained from this ideal case of two brothers, is that sometimes separation between different mice may be more challenging than the separation between different cell types from the same mouse, and that our data is not rich enough to accomplish this task. Another significant point regarding our mice is that their parents are both heterozygotefor Mlh1. This means that in the first stage the mutation rate is much slower and thus the distance in the mutational space between the zygotes of two relatives is very small. All our mice are close relatives of each other, a fact that can explain the somewhat surprising lack of ability to obtain a full separation between different mice, a problem we did not encountered with humans.
